# Strain engineering and bioprocessing strategies for biobased production of porphobilinogen in *Escherichia coli*

**DOI:** 10.1186/s40643-021-00482-3

**Published:** 2021-12-13

**Authors:** Davinder Lall, Dragan Miscevic, Mark Bruder, Adam Westbrook, Marc Aucoin, Murray Moo-Young, C. Perry Chou

**Affiliations:** Department of Chemical Engineering, University of Waterloo, 200 University Avenue West, Waterloo, ON N2L 3G1 Canada

**Keywords:** *Escherichia coli*, Glycerol, Glyoxylate shunt, Porphobilinogen (PBG), Strain engineering, Succinyl-CoA, Tricarboxylic acid (TCA) cycle

## Abstract

Strain engineering and bioprocessing strategies were applied for biobased production of porphobilinogen (PBG) using *Escherichia coli* as the cell factory. The non-native Shemin/C4 pathway was first implemented by heterologous expression of *hemA* from *Rhodopseudomonas spheroids* to supply carbon flux from the natural tricarboxylic acid (TCA) pathways for PBG biosynthesis via succinyl-CoA. Metabolic strategies were then applied for carbon flux direction from the TCA pathways to the C4 pathway. To promote PBG stability and accumulation, Clustered Regularly Interspersed Short Palindromic Repeats interference (CRISPRi) was applied to repress *hemC* expression and, therefore, reduce carbon flowthrough toward porphyrin biosynthesis with minimal impact to cell physiology. To further enhance PBG biosynthesis and accumulation under the *hemC*-repressed genetic background, we further heterologously expressed native *E. coli hemB*. Using these engineered *E. coli* strains for bioreactor cultivation based on ~ 30 g L^−1^ glycerol, we achieved high PBG titers up to 209 mg L^−1^, representing 1.73% of the theoretical PBG yield, with improved PBG stability and accumulation. Potential biochemical, genetic, and metabolic factors limiting PBG production were systematically identified for characterization.

**Graphical Abstract:**

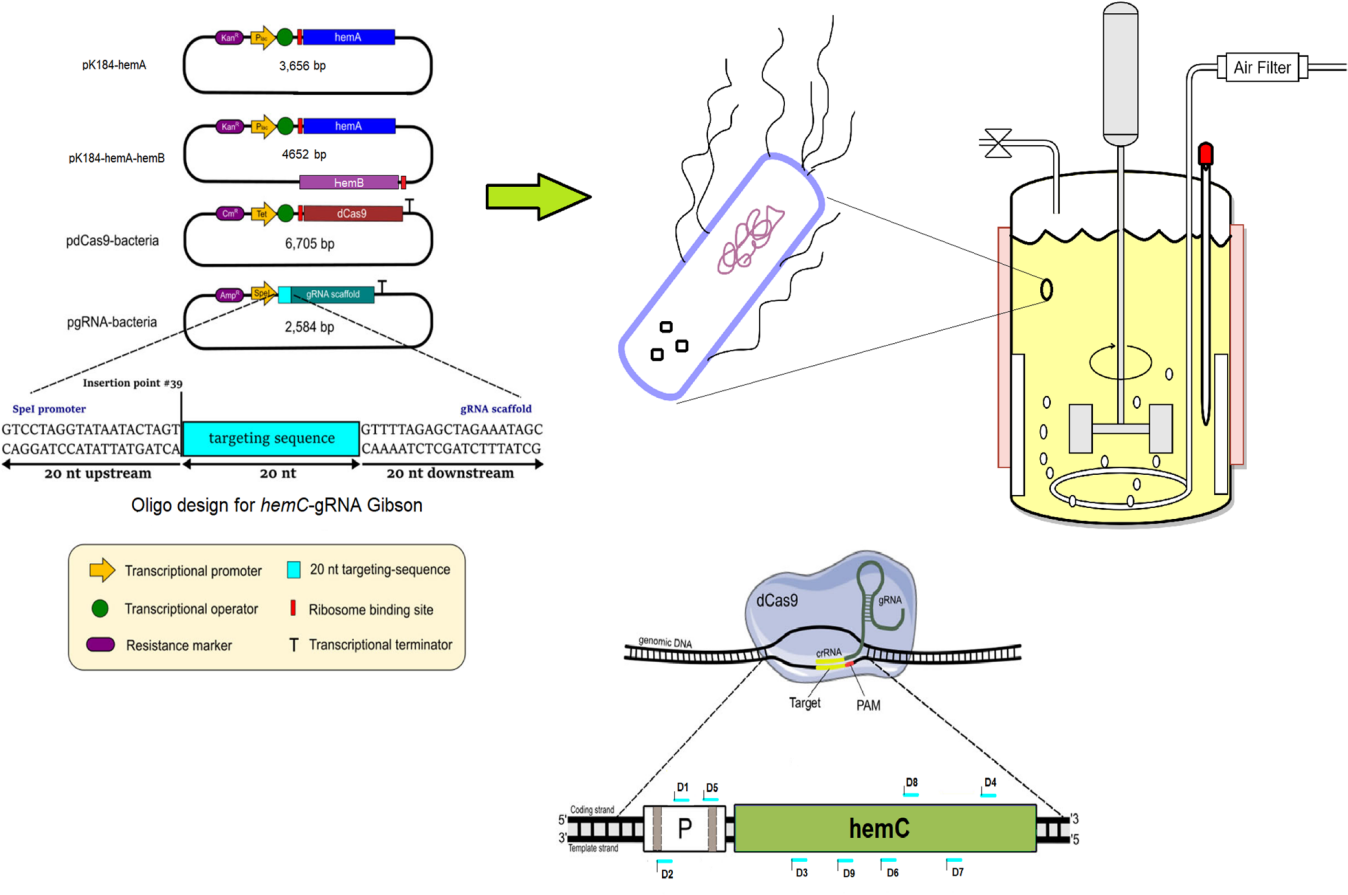

**Supplementary Information:**

The online version contains supplementary material available at 10.1186/s40643-021-00482-3.

## Introduction

Porphobilinogen (PBG) is a pyrrole-containing intermediate in the metabolic pathways for biosynthesis of essential porphyrin/tetrapyrrole compounds known as “pigments of life”, including heme, cobalamin, chlorophyll, siroheme, heme d_1_, etc., in almost all types of biological cells (Frankenberg et al. 2003). For application purposes, PBG can act as a marker for diagnosis of diseases, such as acute intermittent porphyria (Anderson 2019) and lead (Pb) poisoning (Gibson et al. 1968). Naturally in biological systems, the precursor of PBG, i.e., 5-aminolevulinic acid (5-ALA), is synthesized via either of the two unrelated metabolic routes, i.e., the Beale/C5 pathway and the Shemin/C4 pathway (Zhang et al. 2015). Found in most bacteria (including *Escherichia coli*) and all archaea and plants, the C5 pathway starts with the C5-skeleton of glutamate for conducting two enzymic reactions, i.e., initial reduction of glutamyl-tRNA to glutamate-1-semialdehyde (GSA) via NADPH-dependent glutamyl-tRNA reductase (GluTR) and subsequent transamination of GSA via glutamate-1-semialdehyde-2,1-aminomutase (GSAM), to form 5-ALA (Jahn et al. 1992). On other hand, the C4 pathway, present in humans, animals, fungi and the α-group of proteobacteria, involves ALA synthase (ALAS or HemA, encoded by *hemA*) for molecular condensation of succinyl-CoA and glycine to form 5-ALA with the release of carbon dioxide and coenzyme A (CoA) (Nandi 1978). Subsequently, PBG is synthesized via a common reaction for molecular condensation of two 5-ALA molecules catalyzed by ALA dehydratase (ALAD or HemB, encoded by *hemB*) (Layer et al. 2010).

Even with relatively limited applicability up to date, technologies for PBG production have been explored. Chemical synthesis of PBG has been carried out using a variety of precursor molecules, such as diethyl 4-oxopimelate (Jones et al. 1976), 2-methoxy-4-methyl-5-nitropyridine (Frydman et al. 1965), and 2-Hydroxy-4-methyl-5-nitropyridine (Frydman et al. 1969), as well as reaction processes, such as modified synthesis via a porphobilinogen lactam (Kenner et al. 1977), MacDonald’s method (Jackson and MacDonald 1957), and ozonide cleavage reaction (Jacobi and Li 2001). However, these chemical approaches are expensive, time-consuming, complex, and requiring harsh reaction conditions with typically low yields (Neier 2000). While purification of PBG from the urine of patients with acute porphyria is feasible, the producing capacity is knowingly limited (Westall 1952). While biosynthesis of PBG has been alternatively explored in different microbial cell factories, such as *Rhodopseudomonas spheroides* (Hatch and Lascelles 1972b), *E. coli* (Lee et al. 2013), *Chromatium vinosum* (Vogelmann et al. 1975), *Propionibacterium freudenreichii*, etc. (Piao et al. 2004), enhancing such biobased production is considered technically challenging since PBG, as a metabolic intermediate, hardly accumulates.

**Fig. 1:**
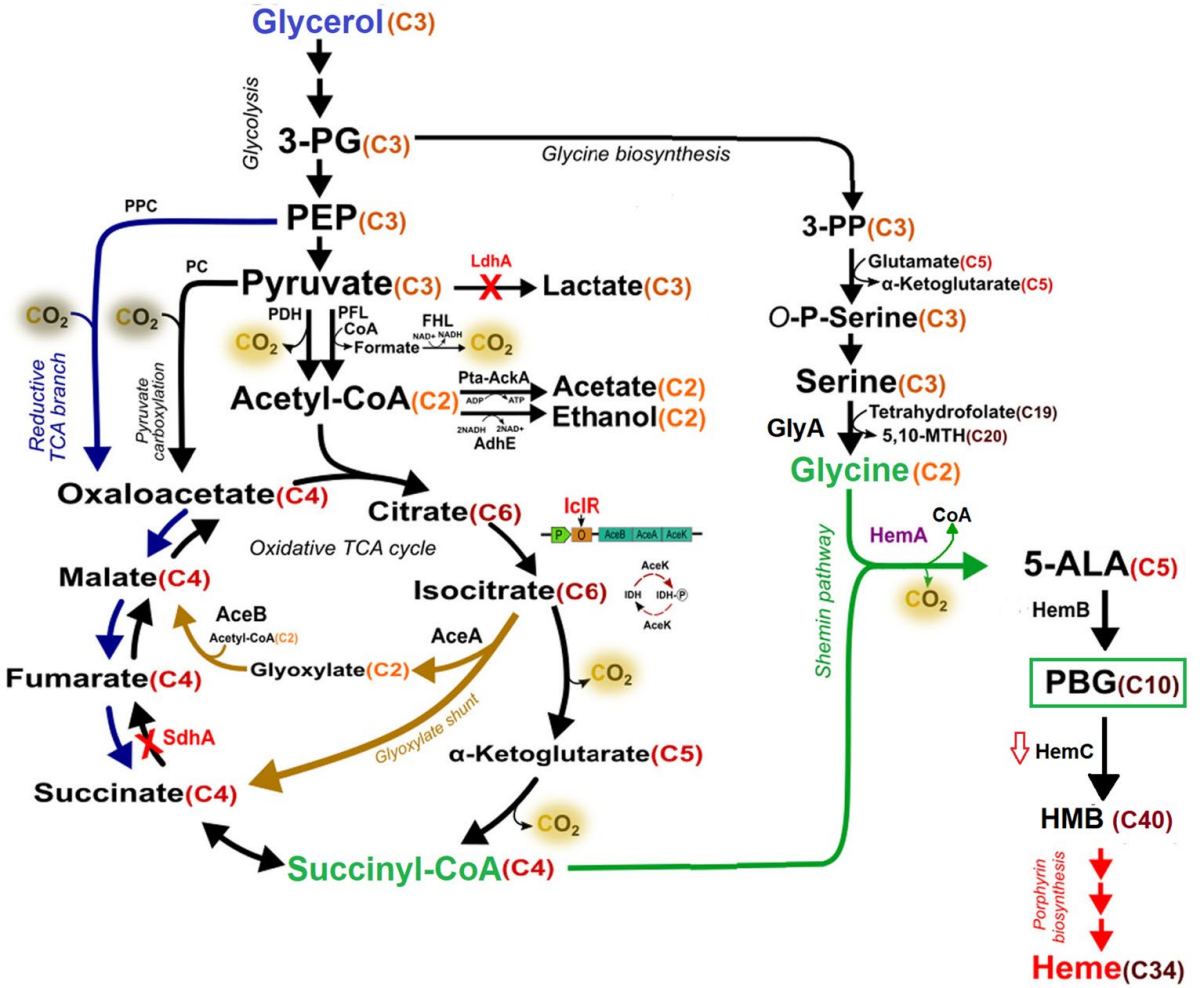
Schematic representation of the natural metabolism and the implemented Shemin pathway for PBG and porphyrin biosynthesis in *E. coli* from glycerol. Metabolic pathways outlined: glycolysis, glycine biosynthesis, pyruvate carboxylation, and oxidative TCA cycle (in black); glyoxylate shunt in the TCA cycle (in light brown); reductive branch of TCA cycle (in blue); Shemin/C4 pathway and its key precursors (in green); porphyrin formation (in red). Colored proteins: mutations (in red); heterologous expression (in purple); carbon source: glycerol (in blue). Metabolite abbreviations: *5,10-MTH* 5,10-methenyltetrahydrofolic acid, *5-ALA* 5-aminolevulinic acid, *3-PG* 3-phosphoglycerate, *3-PP* 3-phosphooxypyruvate, *O-P-Serine* O-phospho-L-serine, *PBG* porphobilinogen; *HMB* hydroxymethylbilane, *PEP* phosphoenolpyruvate, *CoA* coenzyme A. The number of carbon atoms for each metabolite is specified in orange/red. Protein abbreviations: *AceA* isocitrate lyase, *AceB* malate synthase A, *AceK* isocitrate dehydrogenase kinase/phosphatase, *AckA* acetate kinase, *AdhE* aldehyde-alcohol dehydrogenase, *FHL* formate hydrogenlyase, *HemA* 5-aminolevulinate synthase, *HemB* 5-aminolevulinate dehydratase, *HemC* porphobilinogen deaminase, *IclR* AceBAK operon repressor, *IDH* isocitrate dehydrogenase, *IDH-P* isocitrate dehydrogenase-phosphate, *LdhA* lactate dehydrogenase A, *PC* pyruvate carboxylase, *PckA* phosphoenolpyruvate carboxykinase, *PDH* pyruvate dehydrogenase, *PFL* pyruvate formate-lyase, *PK* pyruvate kinase, *PPC* phosphoenolpyruvate carboxylase, *Pta* phosphotransacetylase, *SdhA* succinate dehydrogenase complex (subunit A)

While various cell factories have been developed for biobased production (Chen et al. 2013), bacterium *E. coli* remains the most common one. In native *E. coli,* PBG is synthesized via the C5 pathway and barely accumulates extracellularly since the produced PBG will be readily tetramerized into hydroxymethylbilane (HMB) via porphobilinogen deaminase (PBGD or HemC, encoded by *hemC*) for subsequent biosynthesis of essential porphyrins, such as heme. In this study, we chose to first implement the non-native C4 pathway into *E. coli* for PBG biosynthesis and promote PBG extracellular accumulation, from the structurally unrelated carbon of glycerol by heterologous expression of *hemA* from *R. spheroids* (Fig. 1). Recently, glycerol has been recognized as a promising carbon source for biobased production due to its low cost (Ciriminna et al. 2014), abundancy, and high degree of reduction (Westbrook et al. 2019), resulting in high product yield compared to traditional sugars (Dharmadi et al. 2006). We also developed effective metabolic strategies for carbon flux direction via succinyl-CoA, a key precursor of the C4 pathway. The direction of dissimilated carbon toward succinyl-CoA is dependent on three oxygen-sensitive metabolic routes associated with the central metabolism, i.e., oxidative tricarboxylic acid (TCA) cycle, reductive TCA branch, and glyoxylate shunt (Fig. 1) (Cheng et al. 2013). Under oxygen-deprived (i.e., anaerobic) conditions, succinate (the precursor of succinyl-CoA) acts as an electron acceptor in place of oxygen and accumulates as a final product of mixed acid fermentation via the reductive TCA branch (Thakker et al. 2012). Under oxygen-rich (i.e., aerobic) conditions, succinate acts as a metabolic intermediate of the oxidative TCA cycle without accumulation, but it can also be alternatively derived via the glyoxylate shunt (Thakker et al. 2012). Here, we explored the manipulation of select genes involved in the TCA pathways and cultivation conditions to enhance carbon flux direction into the C4 pathway via succinyl-CoA.

To promote PBG accumulation, we had to limit the activity of subsequent PBG-consuming reactions toward porphyrins. Since porphyrin biosynthesis is essential for cell survival, knocking out any of these PBG-consuming reactions would be lethal (Mobius et al. 2010) (Leung et al. 2021). Hence, we applied Clustered Regularly Interspersed Short Palindromic Repeats interference (CRISPRi) (Qi et al. 2013) to repress the expression of *hemC*, whose encoding gene product of HemC mediates the conversion of PBG to HMB, with minimal impact to cell physiology. To further enhance PBG biosynthesis and accumulation under the *hemC*-repressed genetic background, we also conducted heterologous co-expression of *hemA* from *R. spheroides* and the native *hemB*. In summary, we demonstrated the application of integrated strain engineering and bioprocessing strategies to enhance biosynthesis and ultimate extracellular accumulation of PBG, with systematic identification of potential biochemical, genetic, and metabolic factors limiting PBG production for characterization.

## Materials and methods

### Bacterial strains and plasmids

All bacterial strains and plasmids used in this study are listed in Table 1. Isolation of Genomic DNA from bacterial cells was performed using the Blood & Tissue DNA Isolation Kit (Qiagen, Hilden, Germany). Standard recombinant DNA technologies were applied for molecular cloning (Miller 1992). Phusion and *Taq* DNA polymerase were obtained from New England Biolabs (Ipswich, MA, USA). All synthesized oligonucleotides were ordered from Integrated DNA Technologies (Coralville, IA, USA). DNA sequencing was performed by the Centre for Applied Genomics at the Hospital for Sick Children (Toronto, Canada). *E. coli* BW25113 was the parental strain for derivation of all engineered strains in this study and DH5α was used as an *E. coli* host for molecular cloning. The *ldhA* gene encoding lactate dehydrogenase (LDH) was previously inactivated in BW25113, generating BW∆*ldhA* (Srirangan et al. 2014), a strain with much lower byproduct metabolite production.

**Table 1:** *E. coli* strains and plasmids used in this study

Name	Description or relevant genotype	Source
*E. coli* host strains		
DH5α	F − , *endA1, glnV44, thi-1, recA1, relA1, gyrA96, deoR, nupG φ80d lacZ∆acZd ladlacZYA – argF) U169, hsdR17(rK-mK* +*), λ-*	Lab stock
BW25113	F-, ∆*(araD-araB)567, ∆lacZ4787(::rrnB-3), λ-, rph-1, ∆(rhaD-rhaB)568, hsdR514*	(Datsenko and Wanner 2000)
BW∆*ldhA*	BW25113 *ldhA* null mutant	(Srirangan et al. 2014)
DMH	BW∆*ldhA/*pK-hemA	(Miscevic et al. 2021)
DMH∆*sdhA*	*sdhA* null mutant of DMH	(Miscevic et al. 2021)
DMH∆*iclR*	*iclR* null mutant of DMH	This study
DMH∆*iclR*∆*sdhA*	*iclR* and *sdhA* mutants of DMH	(Miscevic et al. 2021)
DMH-D9∆*sdhA*	DMH∆*sdhA*/pK-hemA/pgRNA-D9/pdcas9-bacteria	This study
DMH-D9∆*iclR*∆*sdhA*	DMH∆*iclR*∆*sdhA* /pK-hemA/pgRNA-D9/pdcas9-bacteria	This study
DSL	BW∆*ldhA/*pK-hemA-hemB	This study
DSL∆*sdhA*	*sdhA* null mutant of DSL	This study
DSL∆*iclR*	*iclR* null mutant of DSL	This study
DSL∆*iclR*∆*sdhA*	*iclR* and *sdhA* mutants of DSL	This study
DSL-D9∆*sdhA*	DSL∆*sdhA*/pK-hemA-hemB/pgRNA-D9/pdcas9-bacteria	This study
DSL-D1∆*iclR*∆*sdhA* DSL-D2∆*iclR*∆*sdhA* DSL-D3∆*iclR*∆*sdhA* DSL-D4∆*iclR*∆*sdhA* DSL-D5∆*iclR*∆*sdhA* DSL-D6∆*iclR*∆*sdhA* DSL-D7∆*iclR*∆*sdhA* DSL-D8∆*iclR*∆*sdhA* DSL-D9∆*iclR*∆*sdhA*	DSL∆*iclR*∆*sdhA* /pK-hemA-hemB/pgRNA-D1/pdcas9-bacteria DSL∆*iclR*∆*sdhA* /pK-hemA-hemB/pgRNA-D2/pdcas9-bacteria DSL∆*iclR*∆*sdhA* /pK-hemA-hemB/pgRNA-D3/pdcas9-bacteria DSL∆*iclR*∆*sdhA* /pK-hemA-hemB/pgRNA-D4/pdcas9-bacteria DSL∆*iclR*∆*sdhA* /pK-hemA-hemB/pgRNA-D5/pdcas9-bacteria DSL∆*iclR*∆*sdhA* /pK-hemA-hemB/pgRNA-D6/pdcas9-bacteria DSL∆*iclR*∆*sdhA* /pK-hemA-hemB/pgRNA-D7/pdcas9-bacteria DSL∆*iclR*∆*sdhA* /pK-hemA-hemB/pgRNA-D8/pdcas9-bacteria DSL∆*iclR*∆*sdhA* /pK-hemA-hemB/pgRNA-D9/pdcas9-bacteria	This study This study This study This study This study This study This study This study This study
Plasmids		
pCP20 pK184	Flp + , λ cI857 + , λ pR Rep(pSC101 ori)ts, ApR, CmR p15A ori, KmR, P*lac::lacZ’*	(Cherepanov and Wackernagel 1995) (Jobling and Holmes 1990)
pdcas9-bacteria	p15A ori, P_Tet_-dCas9	(Qi et al. 2013)
pgRNA-bacteria	ColE1 origin, P_J23119_-gRNA	(Qi et al. 2013)
pgRNA-D1 pgRNA-D2 pgRNA-D3 pgRNA-D4 pgRNA-D5 pgRNA-D6 pgRNA-D7 pgRNA-D8 pgRNA-D9	Derived from pgRNA-bacteria, P_speI_::*hemC-*gRNA-D1 Derived from pgRNA-bacteria, P_speI_::*hemC-*gRNA-D2 Derived from pgRNA-bacteria, P_speI_::*hemC-*gRNA-D3 Derived from pgRNA-bacteria, P_speI_::*hemC-*gRNA-D4 Derived from pgRNA-bacteria, P_speI_::*hemC-*gRNA-D5 Derived from pgRNA-bacteria, P_speI_::*hemC-*gRNA-D6 Derived from pgRNA-bacteria, P_speI_::*hemC-*gRNA-D7 Derived from pgRNA-bacteria, P_speI_::*hemC-*gRNA-D8 Derived from pgRNA-bacteria, P_speI_::*hemC-*gRNA-D9	This study This study This study This study This study This study This study This study This study
pK-hemA	Derived from pK184, P_*lac*_::*hemA*	(Miscevic et al. 2021)
pK-hemA-hemB	Derived from pK184, P_*lac*_::*hemA-hemB*	This study

Genetic implementation of the Shemin/C4 pathway in BW∆*ldhA* was previously described (Miscevic et al. 2021). Heterologous expression of the *hemA* gene cloned in the pK184 vector was under the control of the P_*lac*_ promoter. For heterologous co-expression of *hemA* and *hemB* in BW∆*ldhA*, the native *E. coli hemB* gene was first amplified by polymerase chain reaction (PCR) using the primer set g-hemA-hemB and the genomic DNA of BW∆*ldhA* as the template. The amplified *hemB* gene was Gibson—assembled with PCR-linearized pK184-hemA using the primer set g-pK-hemA-hemB to generate the plasmid pK184-hemA-hemB. Heterologous co-expression of the *hemA* and *hemB* genes cloned in the pK184 vector was also under the control of the P_*lac*_ promoter.

Gene knockouts, including *sdhA* (encoding succinate dehydrogenase (SDH) complex flavoprotein subunit A, SdhA) and *iclR* (encoding transcriptional AceBAK operon repressor, IclR), were introduced into BW∆*ldhA* by P1 phage transduction (Miller 1992) using the appropriate Keio Collection strains (The Coli Genetic Stock Center, Yale University, New Haven, CT, USA) as donors (Baba et al. 2006). For eliminating the co-transduced FRT-Kn^R^-FRT cassette, the transductants were transformed with pCP20 (Cherepanov and Wackernagel 1995), a temperature-sensitive plasmid expressing a flippase (Flp) recombinase. After Flp-mediated excision of the Kn^R^ cassette, a single Flp recognition site (FRT “scar site”) was generated. The pCP20-containing cells were cured by incubation at 42 °C. The genotypes of derived knockout strains were confirmed by colony PCR using the appropriate verification primer sets (Additional file 1: Table S1).

**Fig. 2:**
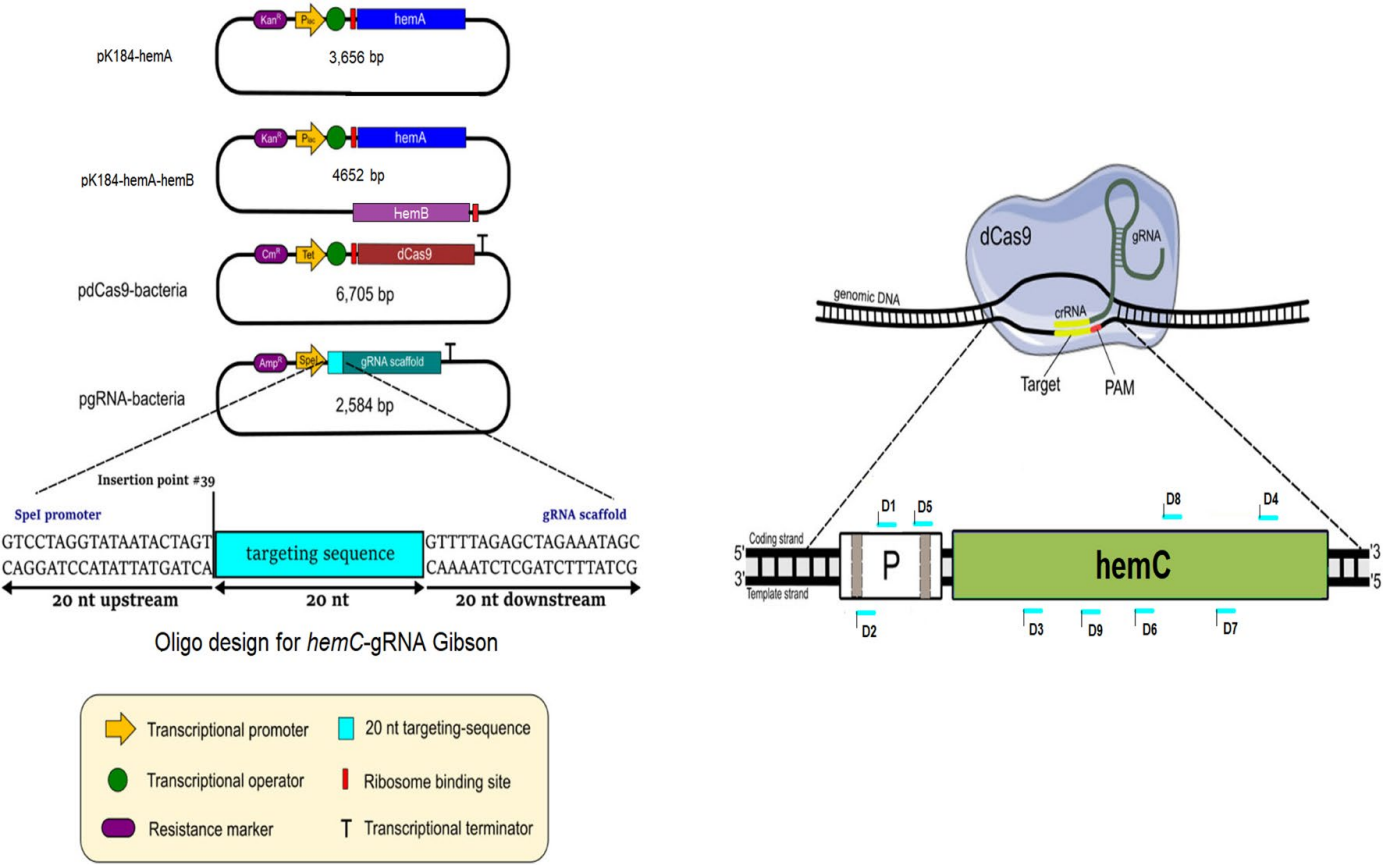
Molecular strategy for CRISPRi-based *hemC* repression. The four plasmids with their major genetic features, such as promoters, antibiotic resistance markers, key genes, for CRISPRi are shown. Select *hemC*-targeting sequences and their associated positions in the *hemC* gene (i.e., D1, D2, D3, D4, D5, D6, D7, D8, and D9) are shown as well. Note that the sequence location, GC content, and predicted *hemC* expression efficiency for various *hemC*-targeting sequences are shown in Additional file 1, in which select *hemC*-repressed strains derived from DSL*∆iclR∆sdhA* were characterized for quantification of the relative *hemC* mRNA level using qRT-PCR compared to the control DSL*∆iclR∆sdhA*. All qRT-PCR experiments were conducted in duplicate

Expression of the *hemC* was repressed by CRISPRi using various derived plasmids from pdcas9-bacteria (Addgene plasmid #44249) and pgRNA-bacteria (Addgene plasmid #44251). The web tool ChopChop (Labun et al. 2016) was used to design sgRNAs with *hemC*-targeting sequences based on predicted expression efficiencies ranging from approximately 20 to 70% (Additional file 1: Table S2). All synthesized oligonucleotide pairs have 60 nucleotides (nt), which include 20 nt *hemC*-targeting sequence, 20 nt upstream and 20 nt downstream sequences of pgRNA-bacteria vector (Fig. 2). They were annealed as described previously (Pengpumkiat et al. 2016), generating double-stranded DNA fragments. These DNA fragments were then individually Gibson-assembled with the PCR-linearized pgRNA-bacteria using the primer set g-pgRNA to generate plasmids, such as pgRNA-D9 (Table 1). The *hemC*-repressed strains can be developed based on a triple-plasmid system (Fig. 2) containing pK184-hemA (or pK184-hemA-hemB), pdcas9-bacteria, and the gRNA-containing plasmid (such as pgRNA-D9).

### Media and bacterial cell cultivation

All medium components were obtained from Sigma-Aldrich Co. (St Louis, MO, USA) except yeast extract and tryptone which were obtained from BD Diagnostic Systems (Franklin Lakes, NJ, USA). *E. coli* strains, stored as glycerol stocks at − 80 °C, were streaked on lysogeny broth (LB; 10 g L^−1^ tryptone, 5 g L^−1^ yeast extract, and 5 g L^−1^ NaCl) agar plates with appropriate antibiotics [ampicillin (100 mg L^−1^), kanamycin (50 mg L^−1^), and chloramphenicol (25 mg L^−1^)] and incubated at 37 °C for 14–16 h.

For shake-flask cultivations, single colonies were picked from LB plates to inoculate 30 mL LB medium in 125-mL conical flasks. The cultures were shaken at 37 °C and 280 rpm in a rotary shaker (New Brunswick Scientific, NJ, USA) and used as seed cultures to inoculate 220 mL LB media at 1% (v/v) in 1-L conical flasks with appropriate antibiotics. This second seed culture was shaken at 37 °C and 280 rpm until the cell density reached 0.80 OD_600_. Cells were then harvested by centrifugation at 9,000 × g and 20 °C for 10 min and resuspended in 30 mL modified M9 production medium. The suspended culture was transferred into 125-mL screwed cap plastic flasks for shaking at 37 °C at 280 rpm in a rotary shaker. Unless otherwise specified, the modified M9 production medium contained 25 g L^−1^ glycerol, 5 g L^−1^ yeast extract, 10 mM NaHCO_3_, 1 mM MgCl_2,_ 200 mL L^−1^ of M9 salts mix (33.9 g L^−1^ Na_2_HPO_4_, 15 g L^−1^ KH_2_PO_4_, 5 g L^−1^ NH_4_Cl, 2.5 g L^−1^ NaCl), 1 mL L^−1^ dilution of Trace Metal Mix A5 (2.86 g L^−1^ H_3_BO_3_, 1.81 g L^−1^ MnCl_2_•4H_2_O, 0.222 g L^−1^ ZnSO_4_•7H_2_O, 0.39 g L^−1^ Na_2_MoO_4_•2H_2_O, 79 µg L^−1^ CuSO_4_•5H_2_O, 49.4 µg L^−1^ Co(NO_3_)_2_•6H_2_O), and was supplemented with 0.1 mM isopropyl β-D-1-thiogalactopyranoside (IPTG).

For bioreactor cultivation, single colonies were picked from LB plates to inoculate 30 mL super broth (SB) medium (32 g L^−1^ tryptone, 20 g L^−1^ yeast extract, and 5 g L^−1^ NaCl) in 125 mL conical flasks. The overnight cultures were shaken at 37 °C and 280 rpm in a rotary shaker (New Brunswick Scientific, NJ, USA) and used as seed cultures to inoculate 220 mL SB media at 1% (v/v) in 1-L conical flasks with appropriate antibiotics. This second seed cultures were shaken at 37 °C and 280 rpm for 14–16 h. Cells were then harvested by centrifugation at 9,000 × g and 20 °C for 10 min and resuspended in 50 mL fresh LB media. The suspended culture was used to inoculate a 1-L stirred tank bioreactor (containing two Rushton radial flow disks as impellers) (CelliGen 115, Eppendorf AG, Hamburg, Germany) at 37 °C and 430 rpm. The semi-defined production medium in the batch bioreactor contained 30 g L^−1^ glycerol, 0.23 g L^−1^ K_2_HPO_4_, 0.51 g L^−1^ NH_4_Cl, 49.8 mg L^−1^ MgCl_2_, 48.1 mg L^−1^ K_2_SO_4_, 1.52 mg L^−1^ FeSO_4_, 0.055 mg L^−1^ CaCl_2_, 2.93 g L^−1^ NaCl, 0.72 g L^−1^ tricine, 10 g L^−1^ yeast extract, 10 mM NaHCO_3_, and 1 mL L^−1^ trace elements (2.86 g L^−1^ H_3_BO_3_, 1.81 g L^−1^ MnCl_2_• 4H_2_O, 0.222 g L^−1^ ZnSO_4_• 7H_2_O, 0.39 g L^−1^ Na_2_MoO_4_• 2H_2_O, 79 μg L^−1^ CuSO_4_• 5H_2_O, 49.4 μg L^−1^ Co(NO_3_)_2_• 6H_2_O) (Neidhardt et al. 1974), and was supplemented with 0.1 mM isopropyl β-D-1-thiogalactopyranoside (IPTG). Aerobic and microaerobic conditions were maintained by purging air into the bulk culture at 1 vvm and into the headspace at 0.1 vvm, respectively. The pH of the media was maintained at 7.0 ± 0.1 with 30% (v/v) NH_4_OH and 15% (v/v) H_3_PO_4_ throughout the bioreactor cultivation.

### Analysis

Culture samples were diluted with 0.15 M saline solution for measuring cell density in OD_600_ using a spectrophotometer (DU520, Beckman Coulter, Fullerton, CA). Cell-free medium (Additional file 1: Table S3) was prepared by centrifugation of the culture sample at 9000 × *g* for 5 min and filter sterilization using a 0.2-µM syringe filter. The quantification of extracellular metabolites and glycerol was conducted using high-performance liquid chromatography (HPLC) (LC-10AT, Shimadzu, Kyoto, Japan) with a refractive index detector (RID; RID-10A, Shimadzu, Kyoto, Japan) and a chromatographic column (Aminex HPX-87H, Bio-Rad Laboratories, CA, USA). The HPLC column temperature was maintained at 35 °C and the mobile phase was 5 mM H_2_SO_4_ (pH 2) running at 0.6 mL min^−1^. The RID signal was acquired and processed by a data processing unit (Clarity Lite, DataApex, Prague, Czech Republic).

PBG titer in the cell-free medium was measured using a regular Ehrlich’s reagent and PBG was colorimetrically quantified by taking an absorbance reading at 555 nm (Mauzerall and Granick 1956). The percentage yield of PBG was defined as the mole ratio of the produced PBG to the theoretically maximal PBG produced based on the consumed glycerol with a molar ratio of one-to-six (i.e., one-mole PBG is derived from six-mole glycerol). Note that one-mole succinyl-CoA (derived from two-mole glycerol) and one-mole glycine (derived from one-mole glycerol) generate one-mole 5-ALA, whereas two-mole 5-ALA forms one-mole PBG. The bulk level of porphyrin compounds in the cell-free medium was estimated using a spectrophotometer at two specific wavelengths, i.e., 405 nm (measuring Soret band) and 495 nm (measuring Q-band). Note that all bioreactor cultivation results shown in this study were, respectively, obtained from a single batch run, with most of cultivation batches being duplicated or even triplicated to ensure their data reproducibility.

### Real-time quantitative reverse transcription PCR (qRT-PCR)

For RNA extraction, *E. coli* cells were cultivated in 30 mL liquid LB medium at 37 °C and harvested in the exponential growth phase. Total RNA isolation was done using the High Pure RNA Isolation Kit (Roche Diagnostics, Basel, Switzerland) as per manufacturer’s instructions and stored at − 80 °C for later analysis. Complementary DNAs (cDNAs) were synthesized from 100 ng of total RNA using the High-Capacity cDNA Reverse Transcription Kit (ThermoFisher Scientific, MA). Sequence-specific primers for *hemC* cDNA (i.e., q-hemC) and internal control *rrsA* (encoding ribosomal RNA 16S) cDNA (i.e., q-rrsA) were used for real-time PCR amplification in 25 µL reaction mixture. qRT-PCR was carried out using the Power SYBR® Green PCR Master Mix (ThermoFisher Scientific; MA) in an Applied Biosystems StepOnePlus™ System as per the manufacturer’s instructions. All quantification experiments were performed in duplicate.

### Statistical analysis

All experimental data in this study were collected in duplicate for statistical analysis. In addition, data comparison was statistically analyzed with an unpaired two-tail Student’s *t*-test based on 95% confidence level to ensure its statistical significance (Additional file 1: Table S4). Hence, *P* < 0.05 was used as a standard criterion of statistical significance when comparing the means of experimental data, such as PBG titer.

## Results

### Carbon flux direction from the TCA pathways to the Shemin/C4 pathway

**Fig. 3:**
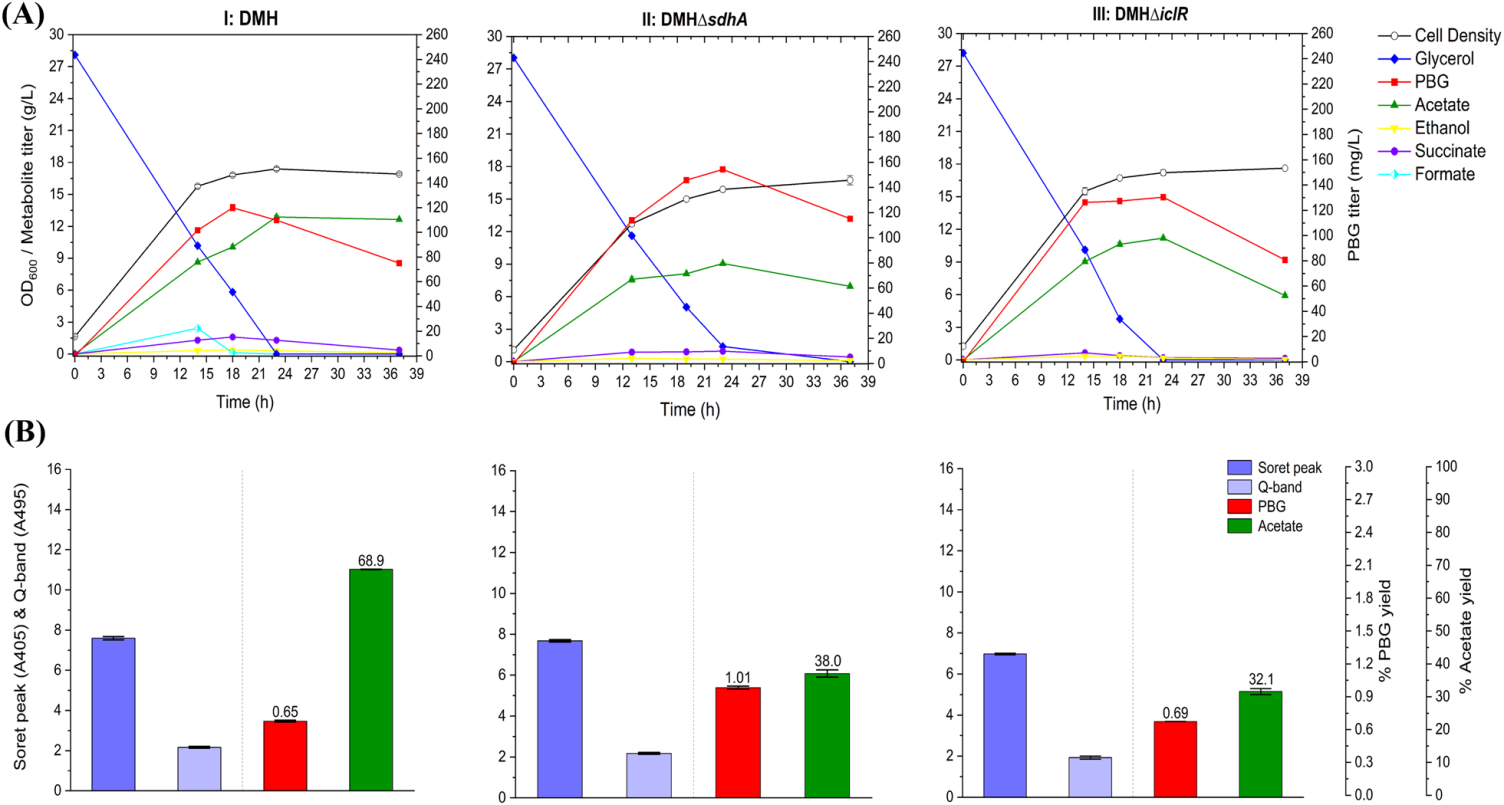
Bioreactor cultivation of DMH, DMH*∆sdhA,* and DMH*∆iclR* for PBG biosynthesis under aerobic conditions. Time profiles of cell density (OD_600_), glycerol consumption and metabolite production profiles, acetate and PBG percentage yields, and extracellular accumulation of porphyrins (represented by the absorbance readings of the Soret peak (A405) and Q-band (A495)) are shown. The percentage yields of acetate/PBG and absorbance readings of porphyrin compounds are calculated/measured based on the consumed glycerol at end of cultivation. (**I**) DMH, (**II**) DMH*∆sdhA,* (**III**) DMH*∆iclR.* All values are reported as means ± SD (*n* = 2)

The Shemin/C4 pathway was implemented in *E. coli* via heterologous expression of *hemA* from *R. sphaeroides* in BW∆*ldhA* (Miscevic et al. 2021). The resulting control strain, DMH, was cultivated under aerobic conditions in a batch bioreactor with ~ 30 g L^−1^ of glycerol as the carbon source. The supply of excess oxygen supported cell growth with effective glycerol consumption, resulting in 120 mg L^−1^ of the peak PBG titer (1.31% yield) with substantial acetate formation (69.3% yield) (Fig. 3). Extending the cultivation, based on the remaining glycerol, and produced acetate, resulted in reduction of PBG titer to 75.1 mg L^−1^ (0.65% yield) with increased porphyrin formation. While the formation of other byproduct metabolites, such as ethanol, succinate, and formate, was minimal, the results suggest the need for metabolic strategies to reduce carbon flux drainage toward acetogenesis and porphyrin biosynthesis for enhanced PBG accumulation. Note that bioreactor characterization was used in this study since shake-flask cultivation resulted in minimal PBG biosynthesis and accumulation (data not shown).

PBG biosynthesis via the C4 pathway utilizes succinyl-CoA as a key precursor (with the other being glycine) to produce 5-ALA as an intermediate before subsequent conversion to PBG. The intracellular succinyl-CoA supply is affected by three oxygen-sensitive metabolic routes associated with the central metabolism, i.e., oxidative TCA cycle, reductive TCA branch, and glyoxylate shunt (Fig. 1). Due to more effective cell growth and glycerol consumption, we first characterized our engineered strains under aerobic conditions. To direct more carbon flux toward the succinyl-CoA node, we inactivate the oxidative TCA cycle by knocking out the *sdhA* gene, resulting in the mutant strain DMH*∆sdhA*, with an improved peak PBG titer of 154 mg L^−1^ (1.41% yield) and 115 mg L^−1^ (1.01% yield) at the end of bioreactor cultivation (Fig. 3). On the other hand, we also deregulated glyoxylate shunt by knocking out the *iclR* gene, resulting in the mutant strain DMH*∆iclR* in which more carbon flux could be directed toward the succinyl-CoA node via glyoxylate shunt with reduced decarboxylation through bypassing the oxidative TCA cycle. Aerobic bioreactor cultivation of DMH*∆iclR* also showed improved peak PBG titer of 130 mg L^−1^ (1.13% yield) and 80.7 mg L^−1^ (0.69% yield) at the end of the cultivation (Fig. 3). Both single-mutant strains of DMH*∆iclR* and DMH*∆sdhA* displayed effective cell growth and glycerol consumption, with reduced acetate production (32.1% and 38.0% yield, respectively) compared to control strain DMH.

**Fig. 4:**
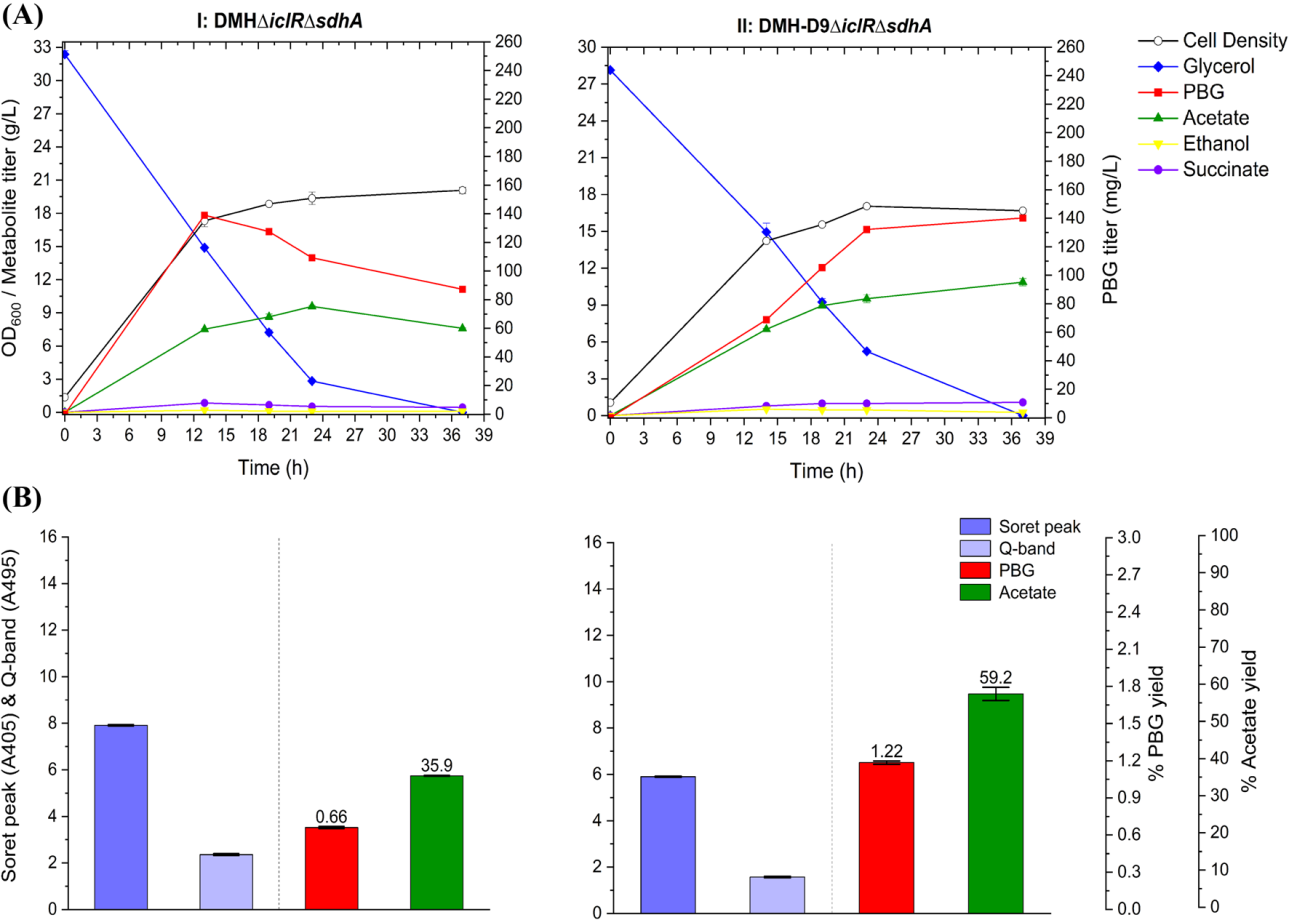
Bioreactor cultivation of DMH*∆iclR∆sdhA* and DMH-D9*∆iclR∆sdhA* for PBG biosynthesis under aerobic conditions. Time profiles of cell density (OD_600_), glycerol consumption and metabolite production profiles, acetate and PBG percentage yields, and extracellular accumulation of porphyrins (represented by the absorbance readings of the Soret peak (A405) and Q-band (A495)) are shown. The percentage yields of acetate/PBG and absorbance readings of porphyrin compounds are calculated/measured based on the consumed glycerol at end of cultivation. Final PBG titer comparison for DMH, DMH*∆iclR∆sdhA,* and DMH-D9*∆iclR∆sdhA* was deemed to be statistically significant (Additional file 1: Table S4). (**I**) DMH*∆iclR∆sdhA*, (**II**) DMH-D9*∆iclR∆sdhA.* All values are reported as means ± SD (*n* = 2)

Next, we derived the double-mutant strain DMH*∆iclR∆sdhA* such that the carbon flux from the deregulated glyoxylate shunt could be further directed toward the succinyl-CoA mode via the reductive TCA branch for enhanced biosynthesis of PBG and porphyrins while minimizing decarboxylation. Aerobic bioreactor cultivation of DMH*∆iclR∆sdhA* produced 87.3 mg L^−1^ (0.66% yield) at the end of cultivation (Fig. 4). Moreover, we observed significantly reduced acetate formation with 35.9% yield, compared to the control strain DMH. These results indicate successful carbon flux direction from the TCA pathways to the C4 pathway in DMH*∆iclR∆sdhA*. However, the directed carbon flux appeared to proceed toward porphyrin formation rather than PBG accumulation in these engineered strains, as indicated by subsequent reduction in PBG titer after reaching a peak value. While blocking the conversion of PBG to HMB by knocking out *hemC* appears to be a feasible way to promote PBG accumulation, such gene knockout is lethal due to physiological requirement of essential porphyrins.

### Repression of* hemC* expression for PBG biosynthesis and accumulation

Since *hemC* is essential for heme biosynthesis, gene knockdown to repress *hemC* expression was explored to promote PBG accumulation with minimal impact on cell physiology and PBG biosynthesis. Hence, CRISPRi was applied using *hemC*-targeting gRNAs with distinct expression efficiencies (predicted by CHOPCHOP). Upon first screening of a selection of gRNAs targeting different areas of *hemC* (Fig. 2; Additional file 1: Table S2) based on bioreactor cultivation, *hemC*-gRNA-D9 appeared to show effective *hemC* repression with enhanced PBG biosynthesis and accumulation. The *hemC*-repression effect was further verified by qRT-PCR (Additional file 1: Figs. S1 and S2). Hence, the resulting *hemC*-repressed strains based on the use of *hemC*-gRNA-D9 were selected for complete bioreactor characterization. Under aerobic bioreactor conditions, cell growth and glycerol utilization for DMH-D9*∆iclR∆sdhA* were minimally affected compared to the control strain DMH*∆iclR∆sdhA*, suggesting that the need of essential porphyrins for cell survival was properly met in the presence of *hemC* repression. Importantly, we observed more effective biosynthesis and accumulation of PBG, achieving a peak/final titer of 140 mg L^−1^ (1.22% yield) at the end of the cultivation (Fig. 4). Note that the Soret peak and Q-band absorbance values of the cell-free medium for the culture sample of DMH-D9*∆iclR∆sdhA* was reduced to some extent, suggesting successful *hemC* repression with reduced porphyrin formation.

### Increasing *hemB* expression to enhance PBG biosynthesis and accumulation

**Fig. 5:**
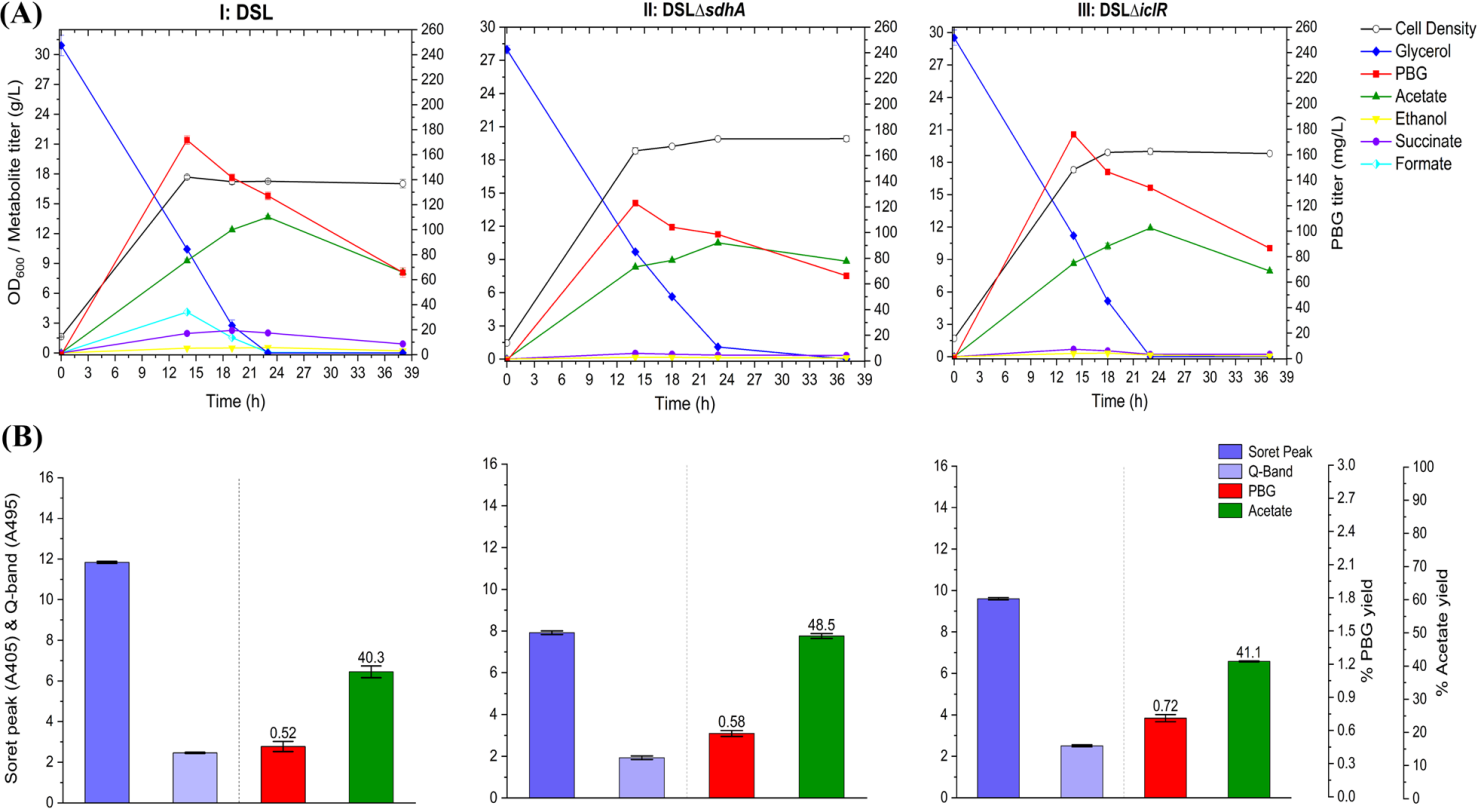
Bioreactor cultivation of DSL, DSL*∆sdhA,* and DSL*∆iclR* for PBG biosynthesis under aerobic conditions. Time profiles of cell density (OD_600_), glycerol consumption and metabolite production profiles, acetate and PBG percentage yields, and extracellular accumulation of porphyrins (represented by the absorbance readings of the Soret peak (A405) and Q-band (A495)) are shown. The percentage yields of acetate/PBG and absorbance readings of porphyrin compounds are calculated/measured based on the consumed glycerol at end of cultivation. (**I**) DSL, (**II**) DSL*∆sdhA,* (**III**) DSL*∆iclR.* All values are reported as means ± SD (*n* = 2)

To further enhance PBG biosynthesis and accumulation, we cloned the native *hemB* gene from *E. coli* for heterologous expression along with *hemA* from *R. sphaeroides*, resulting in another control strain DSL. While aerobic bioreactor cultivation of DSL led to a much higher peak PBG titer compared to DMH, the PBG titer reduced rapidly upon extended cultivation to 65.7 mg L^−1^ (0.52% yield) (Fig. 5), a level similar to DMH. Porphyrin biosynthesis in DSL appeared to be higher than DMH, as evidenced by higher Soret peak and Q-band absorbance values of the cell-free medium for the culture sample. Also note that cell growth and glycerol consumption remained effective for DSL compared to DMH.

**Fig. 6:**
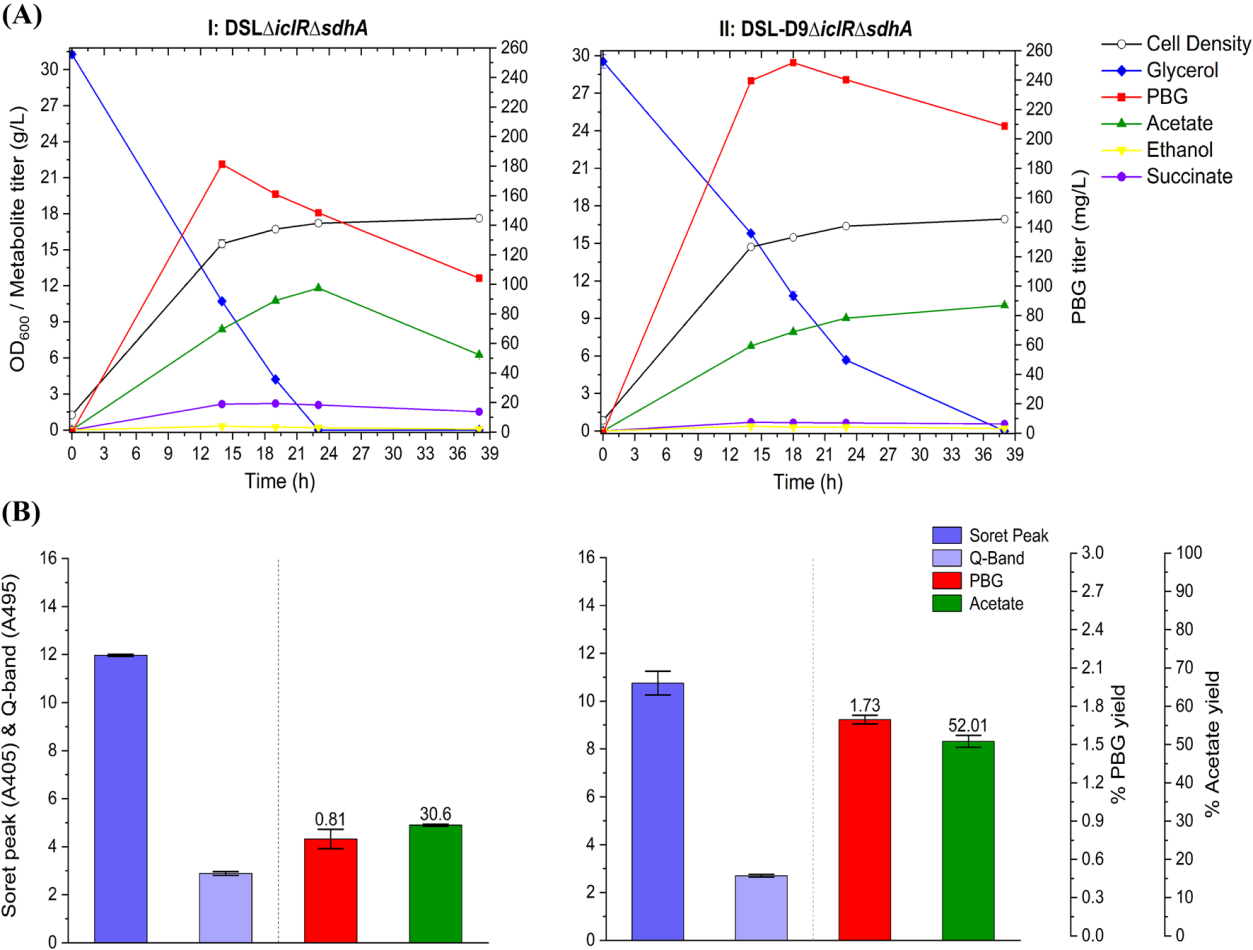
Bioreactor cultivation of DSL*∆iclR∆sdhA* and DSL-D9*∆iclR∆sdhA* for PBG biosynthesis under aerobic conditions. Time profiles of cell density (OD_600_), glycerol consumption and metabolite production profiles, acetate and PBG percentage yields, and extracellular accumulation of porphyrins (represented by the absorbance readings of the Soret peak (A405) and Q-band (A495)) are shown. The percentage yields of acetate/PBG and absorbance readings of porphyrin compounds are calculated/measured based on the consumed glycerol at end of cultivation. Final PBG titer comparison for DSL, DSL*∆iclR∆sdhA,* and DSL-D9*∆iclR∆sdhA* was deemed to be statistically significant (Additional file 1: Table S4). (**I**) DSL*∆iclR∆sdhA*, (**II**) DSL-D9*∆iclR∆sdhA.* All values are reported as means ± SD (*n* = 2)

Similar to DMH, the metabolic limitations associated with excessive carbon flux drainage toward acetogenesis and porphyrin formation in DSL should be addressed. We derived single-mutant strains of DSL*∆sdhA* and DSL*∆iclR* with the *sdhA* and *iclR* gene knockouts, respectively. While these single-mutant strains did not improve PBG biosynthesis significantly upon aerobic bioreactor cultivation, they showed metabolic effects similar to the corresponding DMH single-mutant strains (Fig. 5). We further derived the double-mutant strain DSL*∆iclR∆sdhA*, which showed significantly enhanced PBG biosynthesis compared to the DSL control and single-mutant strains upon aerobic bioreactor cultivation, i.e., a PBG titer of 104 mg L^−1^ (0.81% yield) at the end of the cultivation (Fig. 6). Moreover, reduced acetogenesis was observed in DSL*∆iclR∆sdhA* with effective glycerol utilization and cell growth, suggesting successful carbon flux direction towards the succinyl-CoA node for PBG and porphyrin biosynthesis under this new genetic background.

Furthermore, *hemC*-gRNA-D9 was used to repress *hemC* expression in the double-mutant strain DSL*∆iclR∆sdhA*, resulting in DSL-D9*∆iclR∆sdhA.* Aerobic bioreactor cultivation of DSL-D9*∆iclR∆sdhA* showed much improved PBG biosynthesis and accumulation, i.e., a PBG titer at 209 mg L^−1^ (1.73% yield) at the end of the cultivation, though glycerol consumption and cell growth were slightly affected. Note that the final PBG yield for DSL-D9*∆iclR∆sdhA* was 2.14-fold that for the control DSL*∆iclR∆sdhA*, suggesting the effectiveness of *hemC* repression toward enhanced PBG biosynthesis and accumulation.

### Strain engineering for PBG biosynthesis under microaerobic conditions

**Fig. 7:**
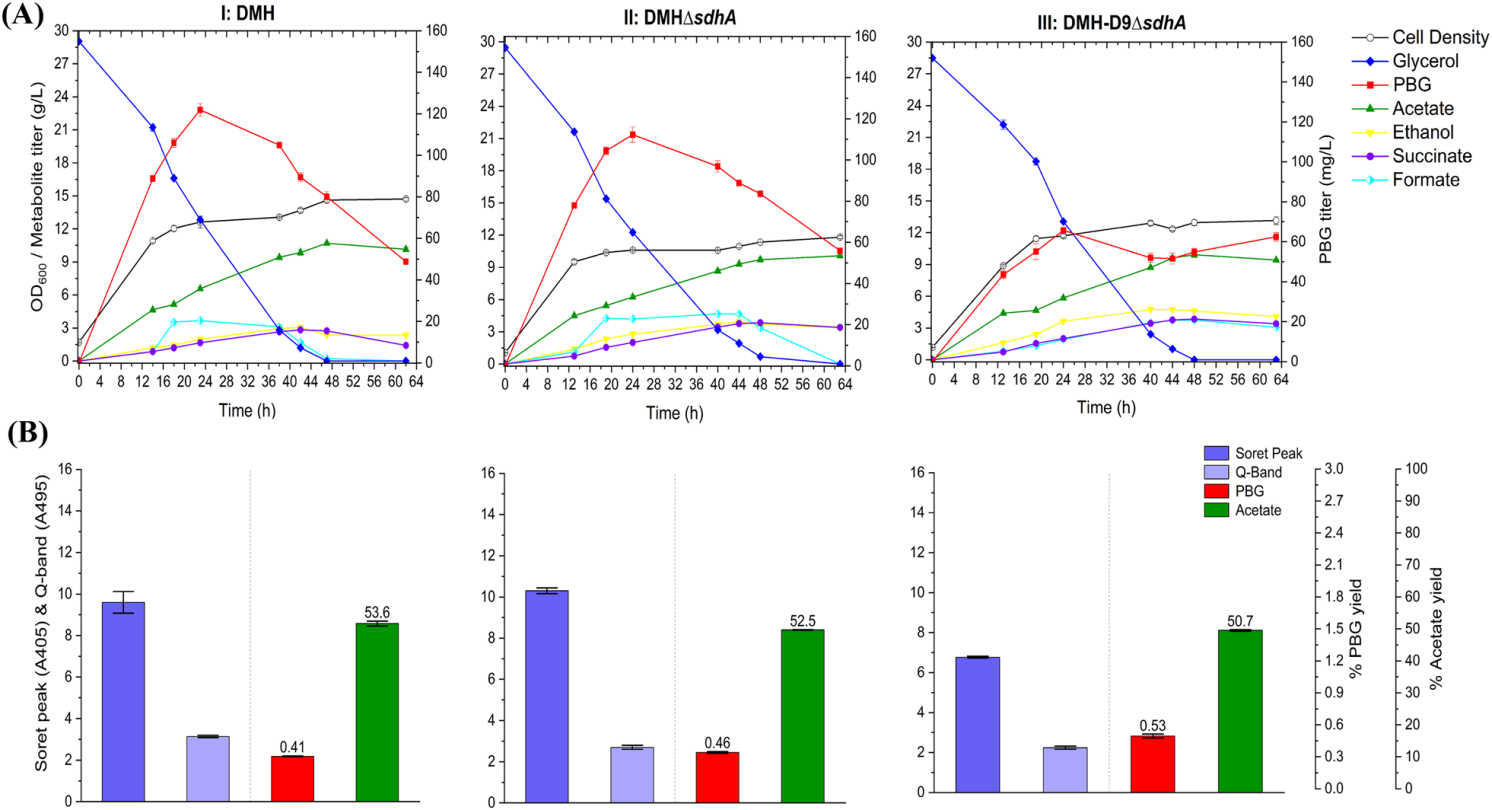
Bioreactor cultivation of DMH, DMH*∆sdhA,* and DMH-D9*∆sdhA* for PBG biosynthesis under microaerobic conditions. Time profiles of cell density (OD_600_), glycerol consumption and metabolite production profiles, acetate and PBG percentage yields, and extracellular accumulation of porphyrins (represented by the absorbance readings of the Soret peak (A405) and Q-band (A495)) are shown. The percentage yields of acetate/PBG and absorbance readings of porphyrin compounds are calculated/measured based on the consumed glycerol at end of cultivation. (**I**) DMH, (**II**) DMH*∆sdhA,* (**III**) DMH-D9*∆sdhA.* All values are reported as means ± SD (*n* = 2)

**Fig. 8:**
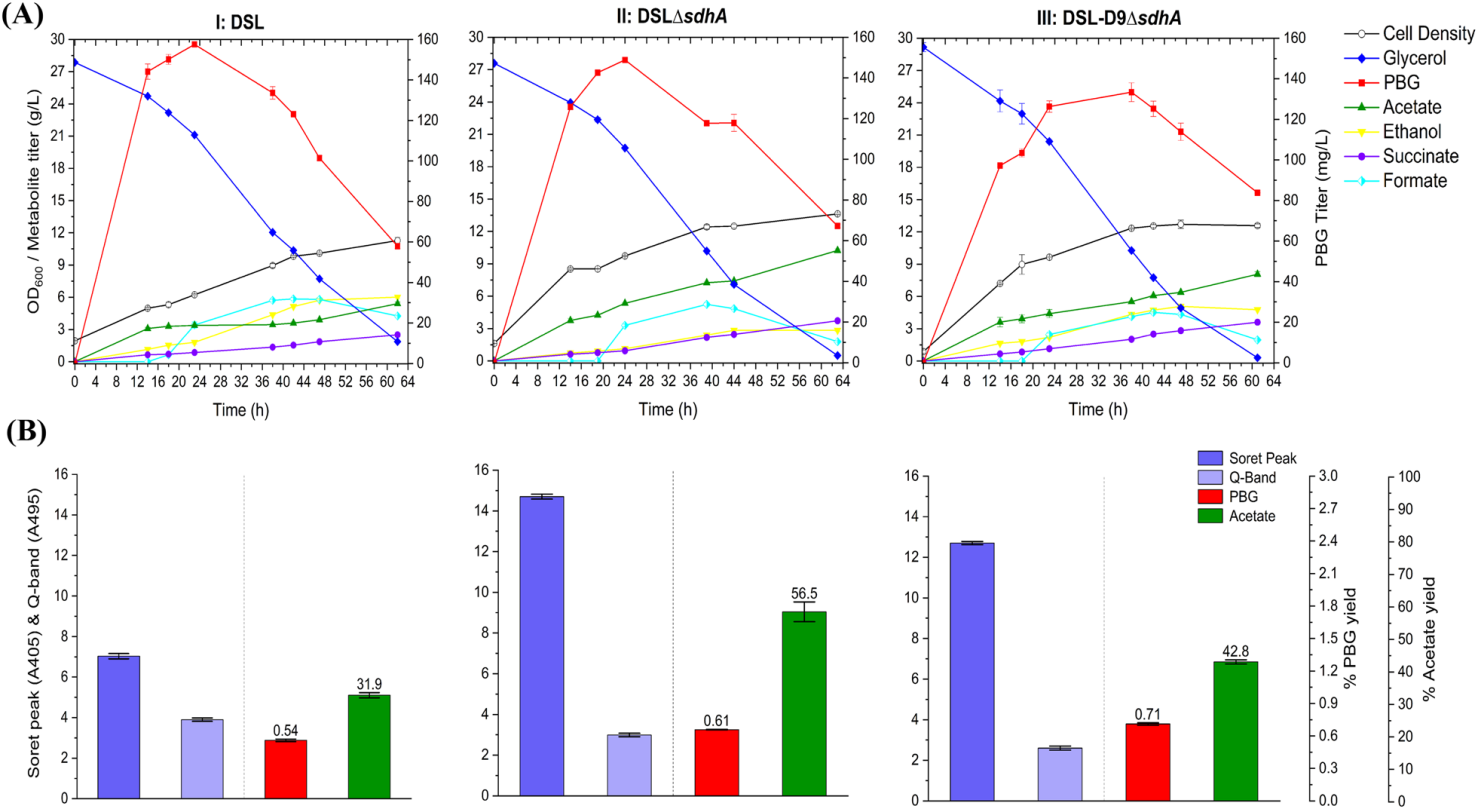
Bioreactor cultivation of DSL, DSL*∆sdhA,* and DSL-D9*∆sdhA* for PBG biosynthesis under microaerobic conditions. Time profiles of cell density (OD_600_), glycerol consumption and metabolite production profiles, acetate and PBG percentage yields, and extracellular accumulation of porphyrins (represented by the absorbance readings of the Soret peak (A405) and Q-band (A495)) are shown. The percentage yields of acetate/PBG and absorbance readings of porphyrin compounds are calculated/measured based on the consumed glycerol at end of cultivation. Final PBG titer comparison for DSL, DSL*∆sdhA,* and DSL-D9*∆sdhA* was deemed to be statistically significant (Additional file 1: Table S4). (**I**) DSL, (**II**) DSL*∆sdhA,* (**III**) DSL-D9*∆sdhA.* All values are reported as means ± SD (*n* = 2)

Using engineered strains with the single *sdhA* mutation, we also explored PBG biosynthesis under oxygen-limited (i.e., microaerobic) conditions. Due to the inactivated oxidative TCA cycle with a regulated glyoxylate shunt, cell growth and glycerol utilization under microaerobic conditions for these control and mutant strains were ineffective compared to aerobic cultivation. In general, PBG biosynthesis under microaerobic conditions was also ineffective compared to aerobic cultivation. For the control strain DMH, the final PBG titer for microaerobic bioreactor cultivation was lower than that for aerobic cultivation, only reaching 48.7 mg L^−1^ (0.41% yield) (Fig. 7), with poor glycerol utilization and cell growth. Interestingly, porphyrin biosynthesis under microaerobic conditions appeared to be more effective, as evidenced by higher Soret peak and Q-band absorbance values, than aerobic cultivation. Compared to the control strain DMH, PBG biosynthesis under microaerobic conditions for the single-mutant strain DMH*∆sdhA*, in which only the reductive TCA branch was functional, was slightly improved, reaching a final PBG titer of 55.9 mg L^−1^ (0.46% yield), with similar acetogenesis, cell growth, glycerol utilization, and porphyrin formation (Fig. 7). We then evaluated the effects of *hemC* repression in DMH-D9*∆sdhA* under microaerobic conditions and observed slightly better PBG biosynthesis, achieving a final PBG titer of 62.5 mg L^−1^ (0.53% yield), with reduced porphyrin formation (Fig. 7). Note that the peak PBG titers for DMH and DMH*∆sdhA* cultivations were comparatively higher than that for DMH-D9*∆sdhA*, implying PBG was rather unstable under such genetic backgrounds. Similar genetic and metabolic effects under microaerobic conditions described above in DMH single-mutant strains were also observed in the corresponding DSL single-mutant strains with higher *hemB* gene dosages. The final PBG titers for microaerobic bioreactor cultivation were 57.9, 67.2, and 83.8 mg L^−1^ for DSL, DSL*∆sdhA*, and DSL-D9*∆sdhA,* respectively (Fig. 8). Note that the PBG yield for DSL-D9*∆sdhA* was only 1.16-fold that for DSL*∆sdhA*, suggesting that the effect of *hemC* repression on PBG biosynthesis and accumulation was insignificant under microaerobic conditions.

## Discussion

As an intermediate in the metabolic pathway for essential porphyrin biosynthesis, PBG barely accumulates and, therefore, can be hardly detected in the extracellular medium upon cultivation of wild-type *E. coli*. In this study, we employed genetic and metabolic strategies for strain engineering of *E. coli* to enhance PBG biosynthesis for extracellular accumulation. First, the Shemin/C4 pathway was genetically implemented in *E. coli* by heterologous expression of *hemA* from *R. sphaeroides* to mediate molecular fusion of succinyl-CoA and glycine to form the key precursor 5-ALA for biosynthesis of PBG and porphyrins. Second, metabolic strategies were applied to direct carbon flux from the TCA pathways to the C4 pathway via the succinyl-CoA node. Third, the metabolic flux within the C4 pathway was further boosted by heterologous co-expression of *hemA* from *R. spheroides* and the native *E. coli hemB*. Finally, CRISPRi was applied to repress *hemC* expression to promote PBG accumulation with minimal impact to cell physiology and viability. PBG biosynthesis and accumulation in various engineered *E. coli* strains were characterized using bioreactor cultivation under different oxygenic (i.e., aerobic and microaerobic) conditions. Note that inclusion of episomal plasmids for heterologous expression of genes and implementing CRISPRi strategy in various engineered strains require constant use of antibiotic selection during cultivation, subsequently increasing the overall production cost.

Compared to the native *E. coli* in which porphyrin biosynthesis was primarily mediated via the C5 pathway, implementation of the heterologous C4 pathway significantly enhanced porphyrin biosynthesis based on visualization of high red-pigmentation upon bacterial cultivation (Additional file 1: Table S3). Nevertheless, PBG titer remained low with significant carbon spill toward acetogenesis, as shown in the control strain DMH cultivated under aerobic conditions. Since succinyl-CoA serves as a key precursor of the C4 pathway for biosynthesis of PBG and porphyrins, metabolic strategies were developed to increase this precursor supply. In *E. coli*, succinyl-CoA can be derived via three oxygen-dependent TCA pathways: (i) reductive TCA branch; (ii) oxidative TCA cycle, and (iii) glyoxylate shunt (Fig. 1) (Cheng et al. 2013). In this study, we explored two metabolic routes for carbon flux direction toward succinyl-CoA within the TCA pathways, i.e., (i) deregulated glyoxylate shunt and reductive TCA branch via the double mutation of *iclR* and *sdhA* under aerobic conditions, and (ii) reductive TCA branch via the single mutation of *sdhA* under microaerobic conditions. Hence, the effects of individual single mutations and double mutation of *iclR* and *sdhA* on PBG biosynthesis were investigated.

Under aerobic conditions, biosynthesis of PBG and porphyrins was enhanced in DMH*∆iclR∆sdhA* compared to the control DMH, suggesting that carbon flux was successfully directed toward succinyl-CoA and then into the C4 pathway. Also, note that acetogenesis was reduced upon involving glyoxylate shunt (which can bypass decarboxylation associated with the oxidative TCA cycle) for carbon flux direction, improving biosynthesis yields for PBG and porphyrins. Nevertheless, a general trend of the time course of PBG titer remained unchanged, i.e., the PBG titer reached a peak value and then declined toward the end of the cultivation. Such PBG instability, potentially caused by unregulated subsequent reactions toward porphyrins, was alleviated by repression of *hemC* expression via CRISPRi in DMH-D9*∆iclR∆sdhA*. PBG (and porphyrin) biosynthesis was further enhanced by heterologous co-expression of *hemA* from *R. spheroides* and the native *E. coli hemB* and, most importantly, all the above metabolic and *hemC*-repression strategies were still functional under this new genetic background, as shown in all corresponding DSL strains. Note that ALA dehydratase (i.e., HemB, encoded by *hemB*) is subject to feedback inhibition by its downstream metabolite of protoporphyrinogen IX (PPIX) (Zhang et al. 2015), potentially limiting the PBG yield. Repression of *hemC* expression could potential reduce PPIX formation and its feedback inhibition on *hemB* expression, and subsequently increase PBG formation. The effects of heterologous expression of *hemB* could be clearly observed by much higher peak and final PBG titers between the corresponding DMH and DSL strains. The effects of amplification of various genes in the porphyrin biosynthetic pathway on porphyrin formation were documented (Lee et al. 2013). Note that, under aerobic culture conditions, the PBG yield of DSL-D9*∆iclR∆sdhA* with all implemented metabolic and genetic strategies was 2.66-fold that of the control DMH.

Under microaerobic conditions, succinyl-CoA was derived primarily via the reductive TCA branch (Shin et al. 2007) and, therefore, the oxidative TCA cycle had to be inactivated, such as mutating *sdhA* in DMH*∆sdhA*, to support functional operation of the central metabolism. While PBG can be produced under microaerobic conditions, bioreactor cultivation suffered poor cell growth and glycerol utilization with significant acetogenesis and PBG instability. Interestingly, porphyrin biosynthesis appeared to be more effective under microaerobic conditions (as evidenced by higher absorbance values for Soret peak and Q-band) than aerobic cultivation though PBG biosynthesis showed the opposite. Compared to aerobic cultivation, significant amounts of formate were observed for PBG-producing strains cultivated under microaerobic conditions, presumably due to the induced activity of pyruvate formate lyase (PFL) under oxygen-limited conditions instead of pyruvate dehydrogenase (PDH) which is mostly active in oxygen-rich environment (Durnin et al. 2009). Adverse effects arising from accumulated formate and acetate on culture performance were reported (Kirkpatrick et al. 2001). Nevertheless, the strain engineering strategies developed for aerobic cultivation, specifically heterologous *hemB* expression and repression of *hemC* expression, were still applicable to microaerobic cultivation though the improving effects were less significant than those under aerobic conditions. Under microaerobic culture conditions, the PBG yield of DSL-D9*∆sdhA* with all implemented strain engineering strategies was 1.73-fold that of the control DMH.

This study has several advantages over other reported PBG biosynthesis studies in variety of microbial systems. We utilized glycerol as cheap feedstock for direct PBG biosynthesis, compared to the process of PBG preparation from 5-ALA by pretreated cells of *Chromatium vinosum* (Vogelmann et al. 1975). We attained a PBG concentration of 0.182 mmol/g-DCW in *E. coli* without extraneous supplementation of succinate and glycine. We obtained maximum PBG concentration of 924 µM compared to 72 µM from *Propionibacterium freudenreichii* (Piao et al. 2004) or 200 µM from *Rhodopseudomonas spheroids* (Hatch and Lascelles 1972b).

## Conclusions

In this study, we demonstrated that implementation of the non-native C4 pathway in *E. coli* was effective to supply carbon flux from the natural TCA pathways for PBG biosynthesis via succinyl-CoA. Metabolic engineering and bioprocessing strategies were further applied for effective carbon flux direction from the TCA pathways to the C4 pathway for enhanced PBG biosynthesis. To promote PBG accumulation, CRISPRi was successfully applied to repress *hemC* expression with minimal impact to cell physiology. The heterologous expression of the native *E. coli hemB* further enhanced overall PBG biosynthesis which was limited by fusion of two 5-ALA molecules catalyzed by HemB. Overall, we enhanced PBG formation and accumulation in engineered *E. coli* by utilizing a cheap carbon source for direct biosynthesis without precursor supplementation. In addition, potential biochemical, genetic, and metabolic factors limiting PBG production were characterized.

## Supplementary Information

**Additional file 1: Table S1**. DNA oligonucleotide sequences used in this study. **Table S2**. gRNA sequences targeting *hemC* for CRISPRi in this study. See Additiona file 1: Figure S1 for qRT-PCR results for select gRNAs. **Table S3**. Tabulated images of bioreactor cultivation samples under aerobic and microaerobic conditions. **Table S4**. Statistical analysis for comparing experimental data of PBG titers. **Figure S1**. Quantification of the relative *hemC* expression for select gRNAs using qRT‐PCR. All qRT‐PCR values are reported as means ± SD (*n* = 2). **Figure S2. **Bioreactor cultivation of DSL-D1*∆iclR∆sdhA*, DSL-D2*∆iclR∆sdhA,* DSL-D3*∆iclR∆sdhA, and* DSL-D4*∆iclR∆sdhA* for PBG biosynthesis under aerobic conditions. Time profiles of cell density (OD_600_), glycerol consumption and metabolite extracellular accumulation profiles are shown. (**I**) DSL-D1*∆iclR∆sdhA*, (**II**) DSL-D2*∆iclR∆sdhA,* (**III**) DSL-D3*∆iclR∆sdhA*, (**IV**) DSL-D4*∆iclR∆sdhA.* All values are reported as means ± SD (n = 2). **Figure S3. **Bioreactor cultivation of DSL-D5*∆iclR∆sdhA*, DSL-D6*∆iclR∆sdhA,* DSL-D7*∆iclR∆sdhA, and* DSL-D8*∆iclR∆sdhA* for PBG biosynthesis under aerobic conditions. Time profiles of cell density (OD_600_), glycerol consumption and metabolite extracellular accumulation profiles are shown. (**I**) DSL-D5*∆iclR∆sdhA*, (**II**) DSL-D6*∆iclR∆sdhA,* (**III**) DSL-D7*∆iclR∆sdhA*, (**IV**) DSL-D8*∆iclR∆sdhA.* All values are reported as means ± SD (*n* = 2).

## Data Availability

Most of data generated or analyzed during this study are included in this published article and its Additional file 1. Additional file 1 data can be made available from the corresponding author upon reasonable request.

## Abbreviations

5-ALA: 5-Aminolevulinic acid
CoA: Coenzyme A
CRISPRi: Clustered Regularly Interspersed Short Palindromic Repeats interference
DCW: Dry cell weight
*E. coli*: *Escherichia coli*
HemA: ALA synthase from *Rhodopseudomonas spheroids* (encoded by *hemA*)
HemB: ALA dehydratase (encoded by *hemB*)
HemC: Porphobilinogen deaminase (PBGD) (encoded by *hemC*)
HMB: Hydroxymethylbilane
IclR: Transcriptional AceBAK operon repressor (encoded by *iclR*)
PBG: Porphobilinogen
rpm: Revolutions per minute
SdhA: Succinate dehydrogenase (SDH) complex flavoprotein subunit A (encoded by *sdhA*)
TCA: Tricarboxylic acid
vvm: Air volume/culture volume/min
