# Supplementary material for: Strain engineering and bioprocessing strategies for biobased production of porphobilinogen in Escherichia coli
Source: Bioresour Bioprocess. 2021 Dec 13;8(1):122. doi: 10.1186/s40643-021-00482-3 (PMC8668860; doi:10.1186/s40643-021-00482-3)

**Additional file 1**

**Title:** Strain engineering and bioprocessing strategies for biobased production of porphobilinogen in *Escherichia coli*

**Authors:** Davinder Lall, Dragan Miscevic, Mark Bruder, Adam Westbrook, Marc Aucoin, Murray Moo-Young, C. Perry Chou*

Department of Chemical Engineering

University of Waterloo

Waterloo, Ontario, Canada N2L 3G1

*Corresponding author:

C. Perry Chou

Department of Chemical Engineering

University of Waterloo

200 University Avenue West

Waterloo, Ontario, Canada N2L 3G1

Email: cpchou@uwaterloo.ca

Telephone: 1-519-888-4567 ext. 33310

| **Primers/oligo name** | **Primer/oligo sequence (Top DNA strand 5’ 🡪 3’)** | **Source** |
| --- | --- | --- |
| v-ldhA | GATAACGGAGATCGGGAATGATTAA;  GGTTTAAAAGCGTCGATGTCCAGTA | (Akawi, Srirangan, Liu, Moo-Young, & Chou, 2015) |
| v-sdhA | CTCTGCGTTCACCAAAGTGT;  ACACACCTTCACGGCAGGAG | (Miscevic et al., 2021) |
| v-iclR | GGTGGAATGAGATCTTGCGA;  CCGACACGCTCAACCCAGAT | (Miscevic et al., 2021) |
| c-frt | AGATTGCAGCATTACACGTCTTGAG;  CCAGCTGCATTAATGAATCGGGCCATGGTCCATATGAATATCCTCC | (Srirangan et al., 2014) |
| c-ptrc  cf-gRNA  q-hemC  q-rrsA  g-hemA  g-pK-hemA  g-hemA-hemB  g-pK-hemA-hemB  cf-pK-hemA  cf-pK-hemA-hemB  g-pgRNA  hemC-gRNA-D1  hemC-gRNA-D2  hemC-gRNA-D3  hemC-gRNA-D4  hemC-gRNA-D5  hemC-gRNA-D6  hemC-gRNA-D7  hemC-gRNA-D8  hemC-gRNA-D9 | CCGATTCATTAATGCAGCTGG;  GGTCTGTTTCCTGTGTGAAATTGTTA  ATCTTTGACAGCTAGCTCAGTCC;  CAAGCTTCAAAAAAAGCACCGA  TCCAGTTTACGTCGCCAGTG;  AATACCCACCGCACCTTGTC  AACTGGAGGAAGGTGGGGAT;  TCACCGTGGCATTCTGATCC  CACAGGAAACAGCTATGACCATGGACTACAATCTGGCACTCGA;  GAGCTCGAATTCGTAATCATTCAGGCAACGACCTCGGC  GCGCCGAGGTCGTTGCCTGAATGATTACGAATTCGAGCTCGGTAC;  AGTGCCAGATTGTAGTCCATGGTCATAGCTGTTTCCTGTGTG  CCGAGGTCGTTGCCTGAATTTCACACAGGAAACAGACCATGACAGACTTAATCC;  ACCGAGCTCGAATTCGTAATCATTTAACGCAGAATCTTCTTCTCAGCC  TTAAGTCTGTCATGGTCTGTTTCCTGTGTGAAATTCAGGCAACGACCTCGG;  CTGAGAAGAAGATTCTGCGTTAAATGATTACGAATTCGAGCTCGGTACC  AACAGCTATGACCATGGACTAC;  ATTCGTAATCATTCAGGCAACG  AACAGCTATGACCATGGACTAC;  TTCGTAATCATTTAACGCAGAAT  GTTTTAGAGCTAGAAATAGCAAGTT;  ACTAGTATTATACCTAGGACTGAGC GTCCTAGGTATAATACTAGTCTCTGGCAAGGATGTTAGGAGTTTTAGAGCTAGAAATAGC; GCTATTTCTAGCTCTAAAACTCCTAACATCCTTGCCAGAGACTAGTATTATACCTAGGAC GTCCTAGGTATAATACTAGTGTTACCGTCATTATCATCCGGTTTTAGAGCTAGAAATAGC; GCTATTTCTAGCTCTAAAACCGGATGATAATGACGGTAACACTAGTATTATACCTAGGAC GTCCTAGGTATAATACTAGTAGACCTTGCGGGAATTCAACGTTTTAGAGCTAGAAATAGC; GCTATTTCTAGCTCTAAAACGTTGAATTCCCGCAAGGTCTACTAGTATTATACCTAGGAC GTCCTAGGTATAATACTAGTAGCTTATTGATGGCGAAATCGTTTTAGAGCTAGAAATAGC; GCTATTTCTAGCTCTAAAACGATTTCGCCATCAATAAGCTACTAGTATTATACCTAGGAC GTCCTAGGTATAATACTAGTACACTGACATCACTCTGGCAGTTTTAGAGCTAGAAATAGC; GCTATTTCTAGCTCTAAAACTGCCAGAGTGATGTCAGTGTACTAGTATTATACCTAGGAC GTCCTAGGTATAATACTAGTCGACGTTGCCGCGCAGGGAGGTTTTAGAGCTAGAAATAGC; GCTATTTCTAGCTCTAAAACCTCCCTGCGCGGCAACGTCGACTAGTATTATACCTAGGAC GTCCTAGGTATAATACTAGTAGAAATCTCGGGTGGCAACGGTTTTAGAGCTAGAAATAGC; GCTATTTCTAGCTCTAAAACCGTTGCCACCCGAGATTTCTACTAGTATTATACCTAGGAC GTCCTAGGTATAATACTAGTCGCCTGAGCAAACTGGATAAGTTTTAGAGCTAGAAATAGC; GCTATTTCTAGCTCTAAAACTTATCCAGTTTGCTCAGGCGACTAGTATTATACCTAGGAC GTCCTAGGTATAATACTAGTTTGGCACTGGCGACGTAAACGTTTTAGAGCTAGAAATAGC; GCTATTTCTAGCTCTAAAACGTTTACGTCGCCAGTGCCAAACTAGTATTATACCTAGGAC | (Srirangan et al., 2014)  (Miscevic et al., 2021)  This study  This study  (Miscevic et al., 2021)  (Miscevic et al., 2021)  This study  This study  This study  This study  (Miscevic et al., 2021)  This study  This study  This study  This study  This study  This study  This study  This study  This study |

**Table S1:** DNA oligonucleotide sequences used in this study

**Notation for oligonucleotides**: v-verification primer, c-cloning primer, cf- confirmation primer, q-reverse transcription primers, and g-Gibson DNA assembly primer. 20 nt *hemC*-targeting sequence is underlined.

**Table S2:** gRNA sequences targeting hemC for CRISPRi in this study. See Figure S1 for qRT-PCR results for select gRNAs.

| **gRNA** | **20 nt targeting sequence** | **sgRNA location on *hemC* gene** | **Predicted % expression efficiency** | **% GC Content** | **RQ for *hemC* expression**  **(From qRT-PCR)** |
| --- | --- | --- | --- | --- | --- |
| *hemC-*gRNA-D1  *hemC-*gRNA-D2  *hemC-*gRNA-D3  *hemC-*gRNA-D4  *hemC-*gRNA-D5  *hemC-*gRNA-D6  *hemC-*gRNA-D7  *hemC-*gRNA-D8  *hemC-*gRNA-D9 | CTCTGGCAAGGATGTTAGGA  GTTACCGTCATTATCATCCG  AGACCTTGCGGGAATTCAAC  AGCTTATTGATGGCGAAATC  ACACTGACATCACTCTGGCA  CGACGTTGCCGCGCAGGGAG  AGAAATCTCGGGTGGCAACG  CGCCTGAGCAAACTGGATAA  TTGGCACTGGCGACGTAAAC | -150  -174  +456  +952  -138  +635  +754  +663  +583 | 43.6  57.9  48.4  39.2  65.4  49.6  65.5  57.9  26.9 | 50  45  50  40  50  75  55  50  55 | -  -  -  -  1.27  0.06  -  -  0.128 |
|  |  |  |  |  |  |

**Table S3:** Tabulated images of bioreactor cultivation samples under aerobic and microaerobic conditions

| **Strain Name** | **0 hr** | **13-14 hr** | **18-19 hr** | **23-24 hr** | **37-39 hr** | **42-44 hr** | **47-48 hr** | **61-63 hr** | **Final PBG titer**  **(mg L^1^)^a^** |  |
| --- | --- | --- | --- | --- | --- | --- | --- | --- | --- | --- |
| **DMH** | 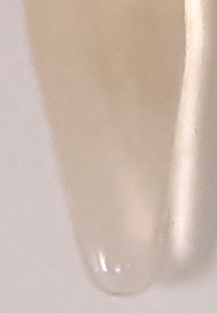 | 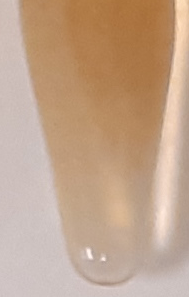 | 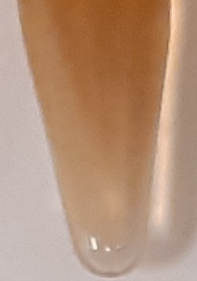 | 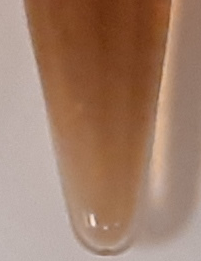 | 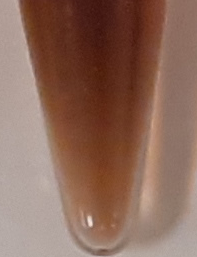 | N/A | | | 75.1 ± 1.5  (0.65%) | Aerobic |
| **DMH∆*sdhA*** | 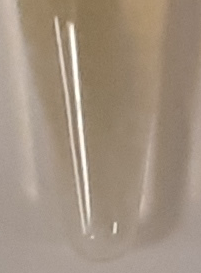 | 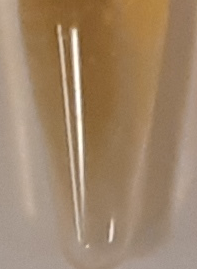 | 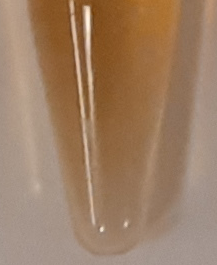 | 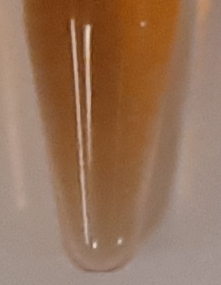 | 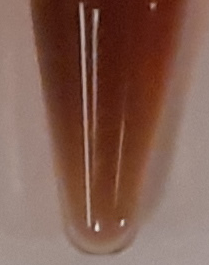 |  |  |  | 115 ± 2.0  (1.01%) |  |
| **DMH∆*iclR*** | 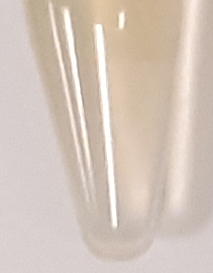 | 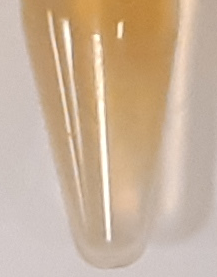 | 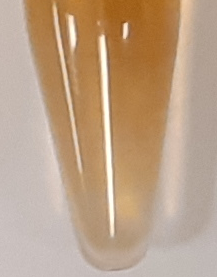 | 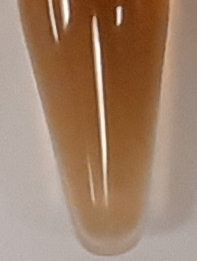 | 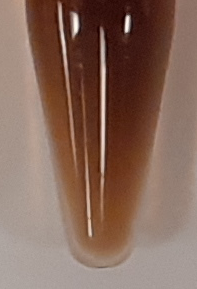 |  |  |  | 80.7 ± 0.4  (0.69%) |  |
| **DMH∆*iclR*∆*sdhA*** | 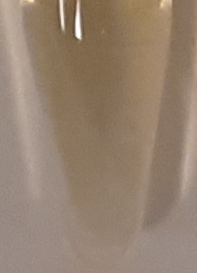 | 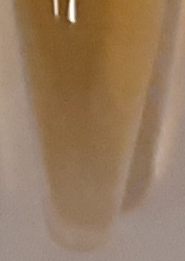 | 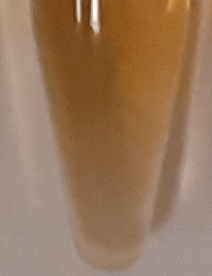 | 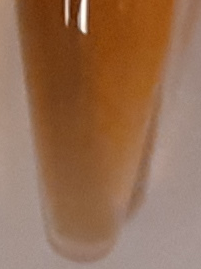 | 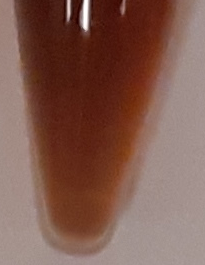 |  |  |  | 87.3 ± 0.3  (0.66%) |  |
| **DMH-D9∆*iclR*∆*sdhA*** | 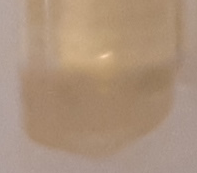 | 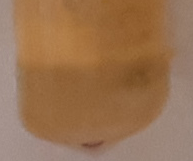 | 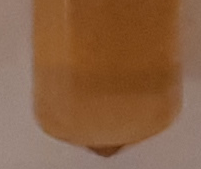 | 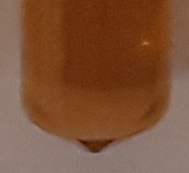 | 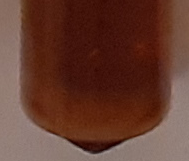 |  |  |  | 140.2 ± 1.8 (1.22%) |  |
| **DSL** | 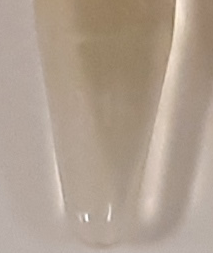 | 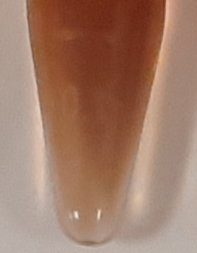 | 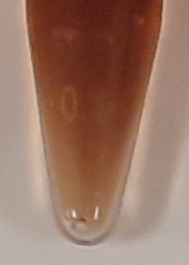 | 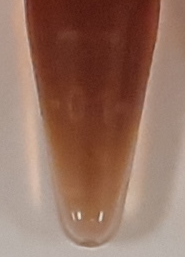 | 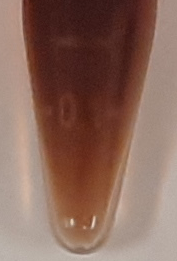 |  |  |  | 65.7 ± 3.7  (0.52%) |  |
| **DSL∆*sdhA*** | 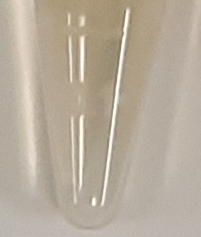 | 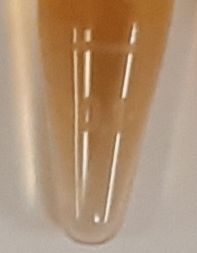 | 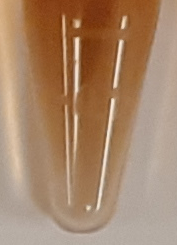 | 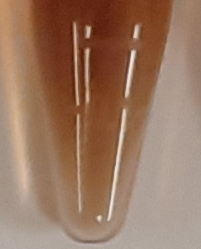 | 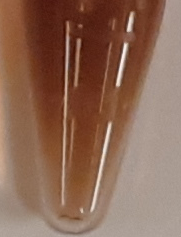 |  |  |  | 66.3 ± 2.2  (0.58%) |  |
| **DSL∆*iclR*** | 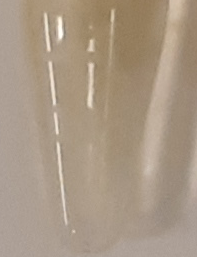 | 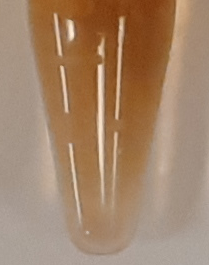 | 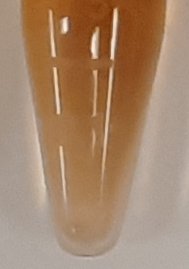 | 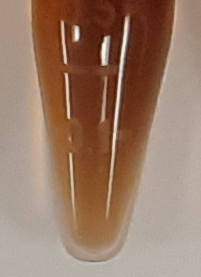 | 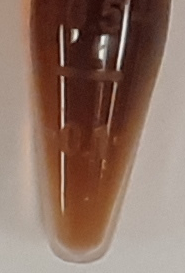 |  |  |  | 86.6 ± 0.1  (0.72%) |  |
| **DSL∆*iclR*∆*sdhA*** | 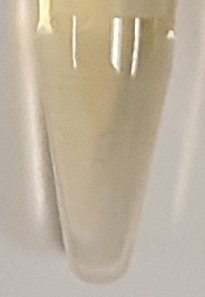 | 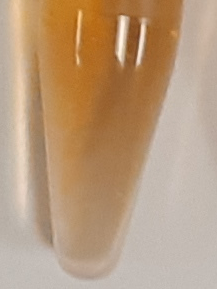 | 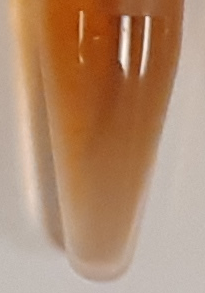 | 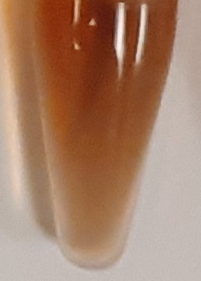 | 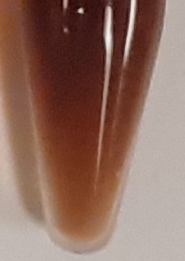 |  |  |  | 104 ± 1.4  (0.81%) |  |
| **DSL-D9∆*iclR*∆*sdhA*** | 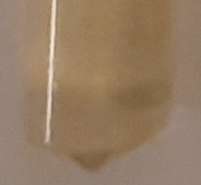 | 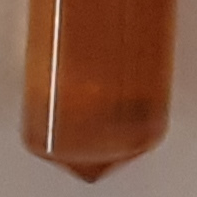 | 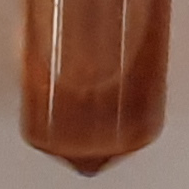 | 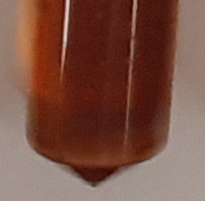 | 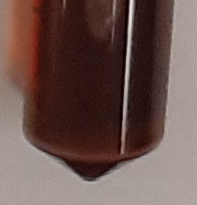 |  |  |  | 209 ± 0.3  (1.73%) |  |
| **DMH** | 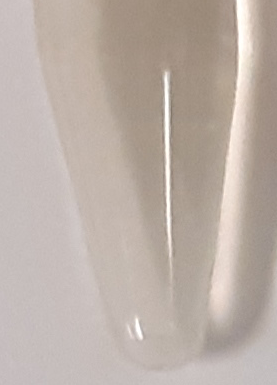 | 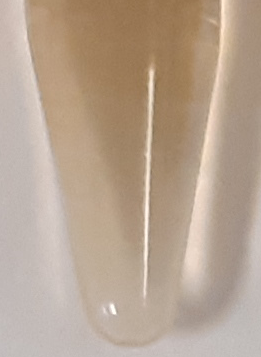 | 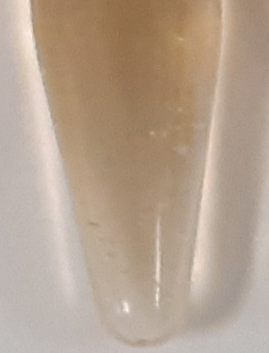 | 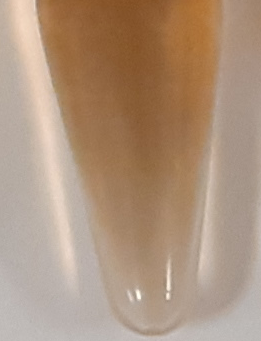 | 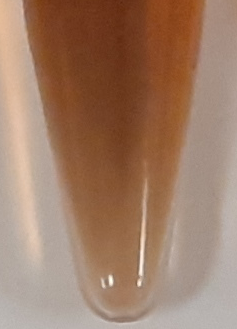 | 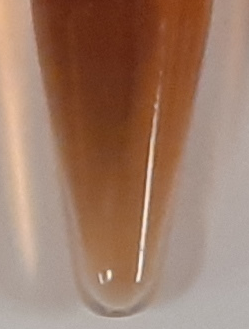 | 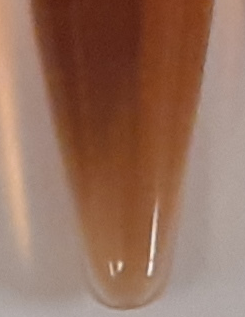 | 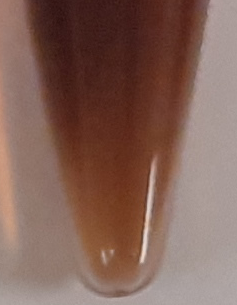 | 48.7 ± 0.6  (0.41%) | Micro-aerobic |
| **DMH∆*sdhA*** | 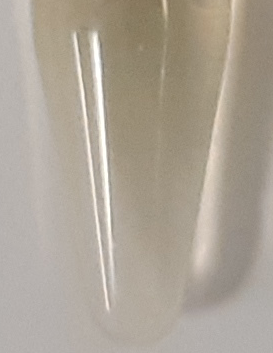 | 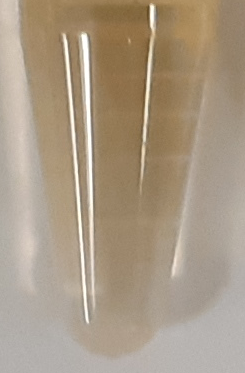 | 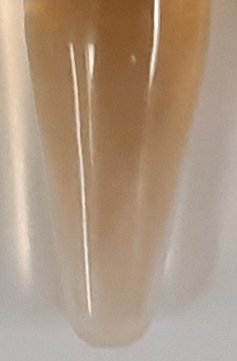 | 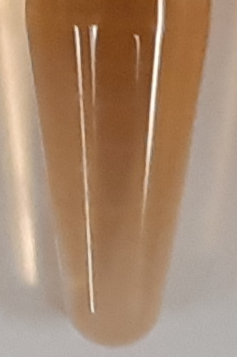 | 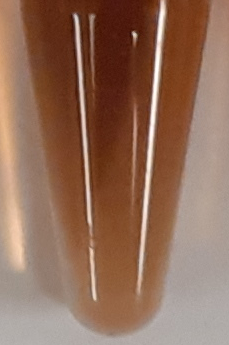 | 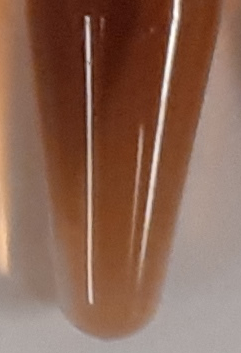 | 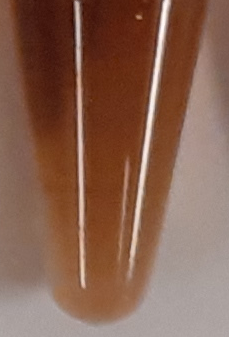 | 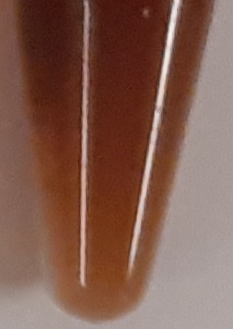 | 55.9 ± 0.6  (0.46%) |  |
| **DMH-D9∆*sdhA*** | 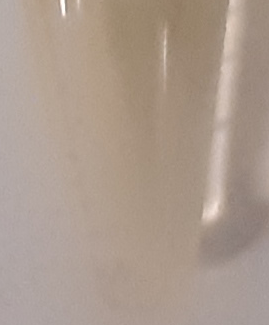 | 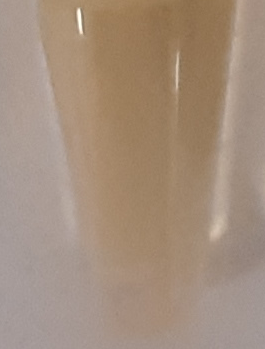 | 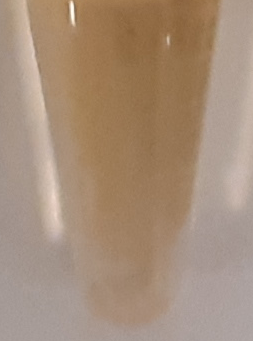 | 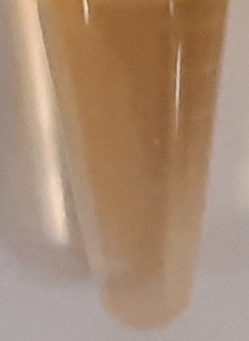 | 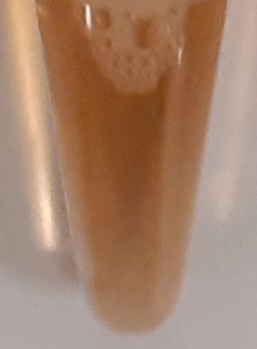 | 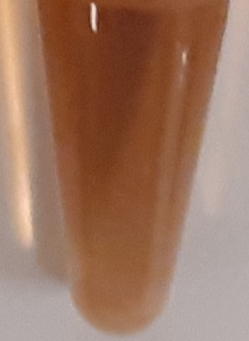 | 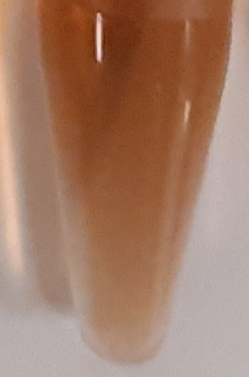 | 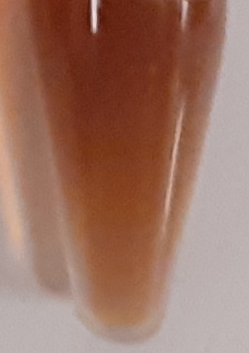 | 62.5 ± 2.1  (0.53%) |  |
| **DSL** | 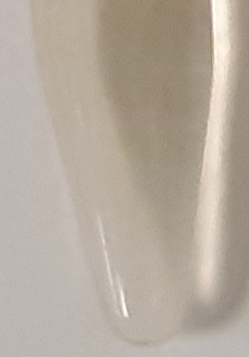 | 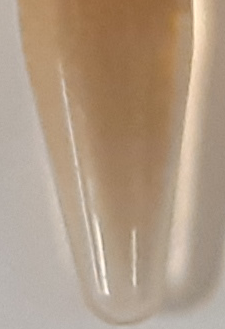 | 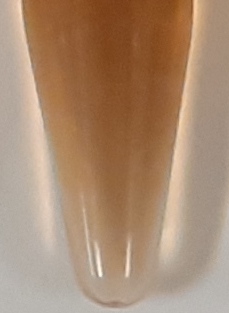 | 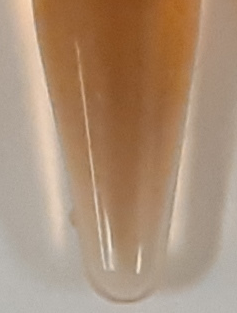 | 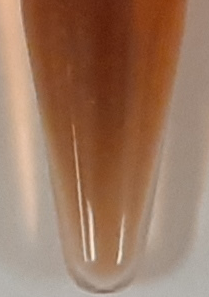 | 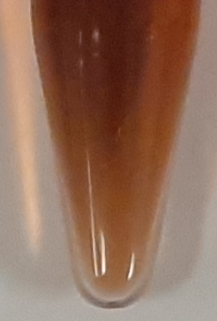 | 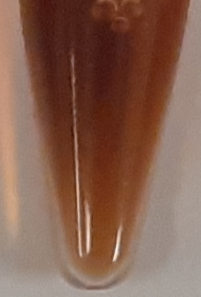 | 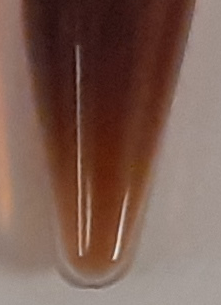 | 57.9 ± 1.2  (0.54%) |  |
| **DSL∆*sdhA*** | 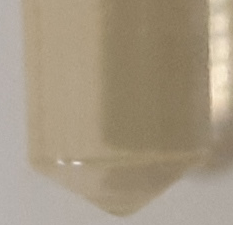 | 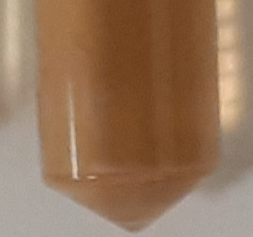 | 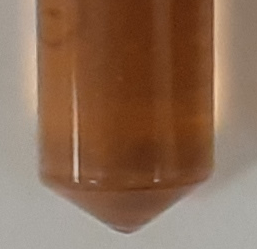 | 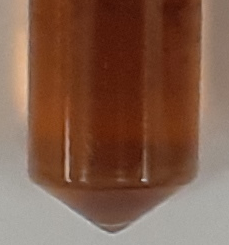 | 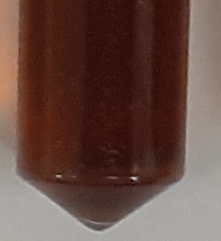 | 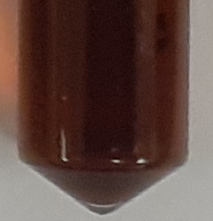 | N/A | 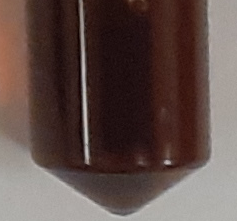 | 67.2 ± 0.9  (0.61%) |  |
| **DSL-D9∆*sdhA*** | 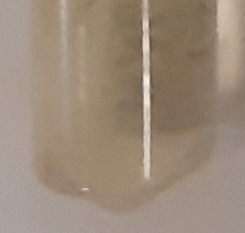 | 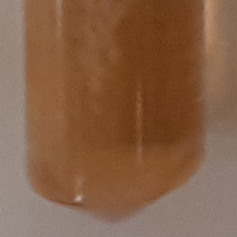 | 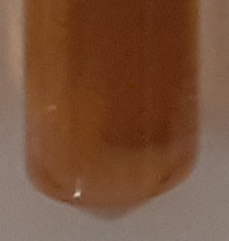 | 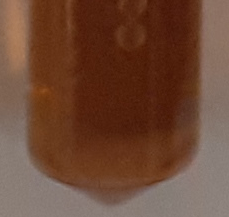 | 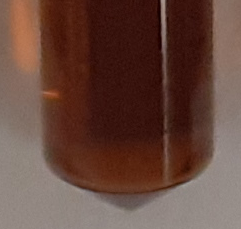 | 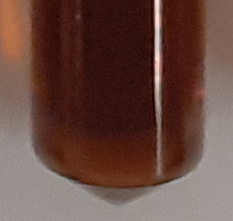 | 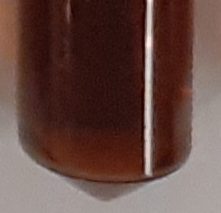 | 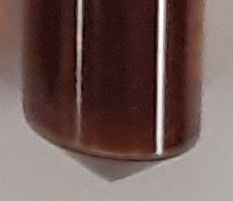 | 83.8 ± 0.2  (0.71) |  |

^a^ PBG extracellular accumulation (mg L^-1^) after complete consumption of glycerol, theoretical percent yield based on consumed glycerol is presented in parentheses

N/A not applicable

**Table S4:** Statistical analysis for comparing experimental data of PBG titers

| **Group #1** | | **Group #2** | | ***P*-value** | **Statistical significance** |
| --- | --- | --- | --- | --- | --- |
| **Strain name** | **PBG**  **(mg L^-1^)** | **Strain name** | **PBG**  **(mg L^-1^)** |  |  |
| **Aerobic Conditions** | | | | | |
| DMH | 75.1 ± 1.5 | DMH∆*sdhA* | 115 ± 2.0 | 0.0015 | *P* < 0.05^*^ |
| DMH | 75.1 ± 1.5 | DMH∆*iclR* | 80.7 ± 0.4 | 0.104 | *P* > 0.05 |
| DMH | 75.1 ± 1.5 | DMH∆*iclR*∆*sdhA* | 87.3 ± 0.3 | 0.049 | *P* < 0.05^*^ |
| DMH | 75.1 ± 1.5 | DMH-D9∆*iclR*∆*sdhA* | 140.2 ± 1.8 | 0.0008 | *P* < 0.05^*^ |
| DMH∆*iclR*∆*sdhA* | 87.3 ± 0.3 | DMH-D9∆*iclR*∆*sdhA* | 140.2 ± 1.8 | 0.0132 | *P* < 0.05^*^ |
| DSL | 65.7 ± 3.7 | DSL∆*sdhA* | 66.3 ± 2.2 | 0.860 | *P* > 0.05 |
| DSL | 65.7 ± 3.7 | DSL∆*iclR* | 86.6 ± 0.1 | 0.041 | *P* < 0.05^*^ |
| DSL | 65.7 ± 3.7 | DSL∆*iclR*∆*sdhA* | 104 ± 1.4 | 0.024 | *P* < 0.05^*^ |
| DSL | 65.7 ± 3.7 | DSL-D9∆*iclR*∆*sdhA* | 209 ± 0.3 | 0.011 | *P* < 0.05^*^ |
| DSL∆*iclR*∆*sdhA* | 104 ± 1.4 | DSL-D9∆*iclR*∆*sdhA* | 209 ± 0.3 | 0.004 | *P* < 0.05^*^ |
| DMH | 75.1 ± 1.5 | DSL | 65.7 ± 3.7 | 0.133 | *P* > 0.05 |
| DMH*∆iclR*∆*sdhA* | 87.3 ± 0.3 | DSL∆*iclR*∆*sdhA* | 104 ± 1.4 | 0.030 | *P* < 0.05^*^ |
| DMH | 75.1 ± 1.5 | DSL-D9∆*iclR*∆*sdhA* | 209 ± 0.3 | 0.003 | *P* < 0.05^*^ |
| **Microaerobic Conditions** | | | | | |
| DMH | 48.7 ± 0.6 | DMH∆*sdhA* | 55.9 ± 0.6 | 0.007 | *P* < 0.05^*^ |
| DMH | 48.7 ± 0.6 | DMH-D9∆*sdhA* | 62.5 ± 2.1 | 0.053 | *P* > 0.05 |
| DMH∆*sdhA* | 55.9 ± 0.6 | DMH-D9∆*sdhA* | 62.5 ± 2.1 | 0.122 | *P* > 0.05 |
| DSL | 57.9 ± 1.2 | DSL∆*sdhA* | 67.2 ± 0.9 | 0.017 | *P* < 0.05^*^ |
| DSL | 57.9 ± 1.2 | DSL-D9∆*sdhA* | 83.8 ± 0.2 | 0.018 | *P* < 0.05^*^ |
| DSL∆*sdhA* | 67.2 ± 0.9 | DSL-D9∆*sdhA* | 83.8 ± 0.2 | 0.018 | *P* < 0.05^*^ |
| DMH | 48.7 ± 0.6 | DSL | 57.9 ± 1.2 | 0.027 | *P* < 0.05^*^ |
| DMH∆*sdhA* | 55.9 ± 0.6 | DSL∆*sdhA* | 67.2 ± 0.9 | 0.006 | *P* < 0.05^*^ |
| DMH | 48.7 ± 0.6 | DSL-D9∆*sdhA* | 83.8 ± 0.2 | 0.004 | *P* < 0.05^*^ |

^*^ Difference between two means is considered to be statistically significant

**Figure S1:** Quantification of the relative *hemC* expression for select gRNAs using qRT‐PCR. All qRT‐PCR values are reported as means ± SD (n = 2).


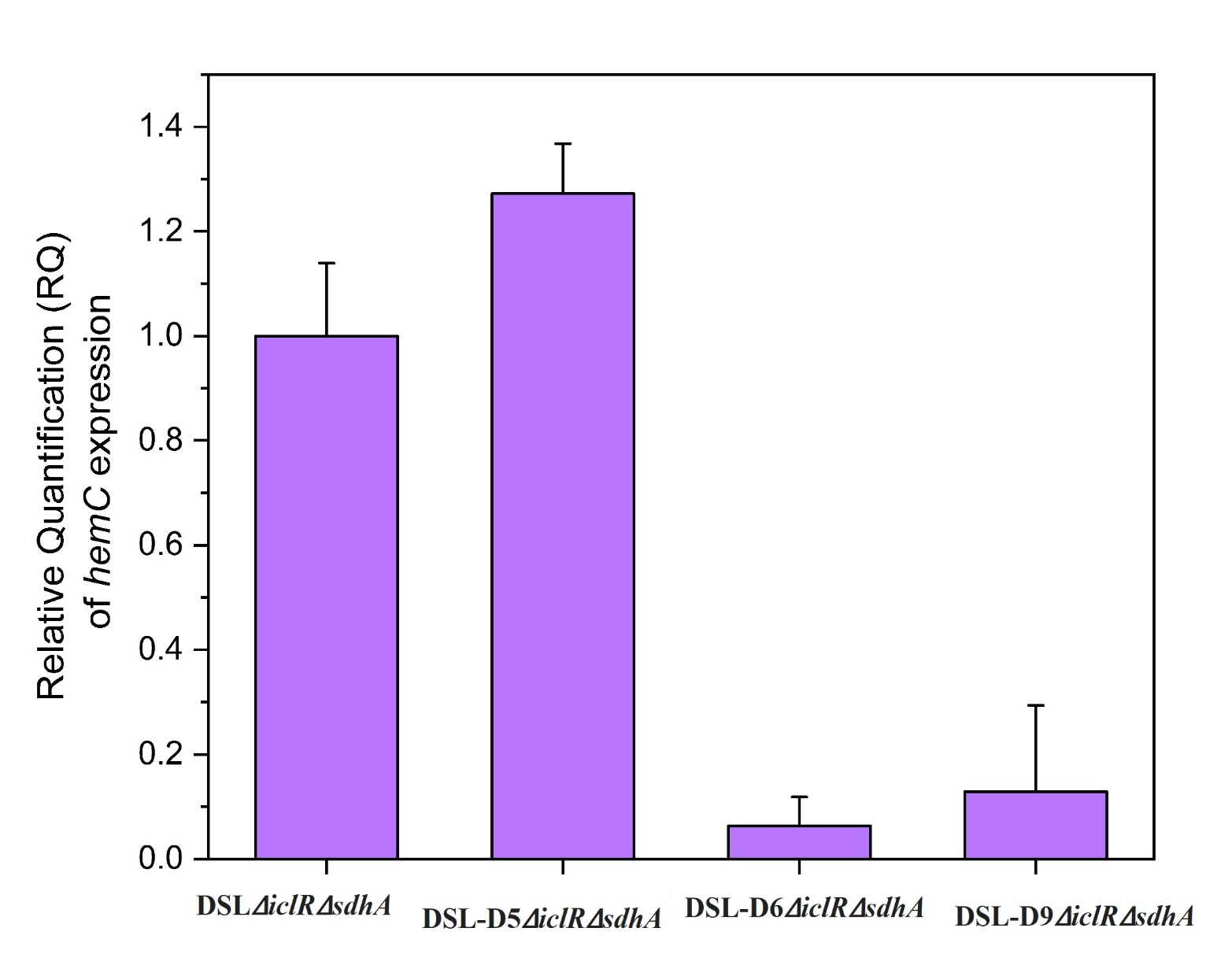


**Figure S2: Bioreactor cultivation of DSL-D1*∆iclR∆sdhA*, DSL-D2*∆iclR∆sdhA,* DSL-D3*∆iclR∆sdhA, and* DSL-D4*∆iclR∆sdhA* for PBG biosynthesis under aerobic conditions.** Time profiles of cell density (OD_600_), glycerol consumption and metabolite extracellular accumulation profiles are shown. (**I**) DSL-D1*∆iclR∆sdhA*, (**II**) DSL-D2*∆iclR∆sdhA,* (**III**) DSL-D3*∆iclR∆sdhA*, (**IV**) DSL-D4*∆iclR∆sdhA.* All values are reported as means ± SD (n = 2).


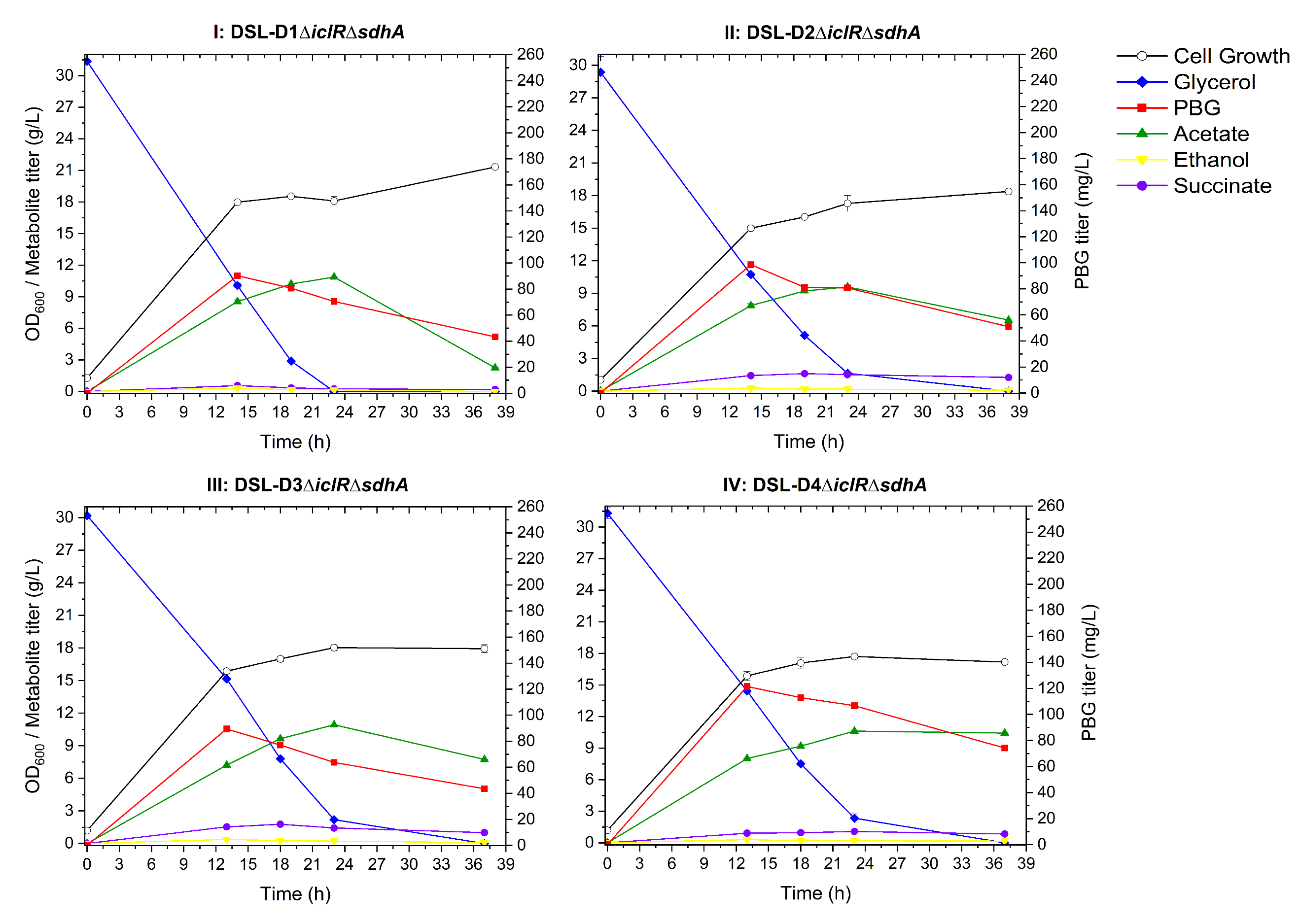


**Figure S3: Bioreactor cultivation of DSL-D5*∆iclR∆sdhA*, DSL-D6*∆iclR∆sdhA,* DSL-D7*∆iclR∆sdhA, and* DSL-D8*∆iclR∆sdhA* for PBG biosynthesis under aerobic conditions.** Time profiles of cell density (OD_600_), glycerol consumption and metabolite extracellular accumulation profiles are shown. (**I**) DSL-D5*∆iclR∆sdhA*, (**II**) DSL-D6*∆iclR∆sdhA,* (**III**) DSL-D7*∆iclR∆sdhA*, (**IV**) DSL-D8*∆iclR∆sdhA.* All values are reported as means ± SD (n = 2).

Akawi, L., Srirangan, K., Liu, X., Moo-Young, M., & Chou, C. (2015). Engineering Escherichia coli for high-level production of propionate. *Journal of Industrial Microbiology & Biotechnology, 42*. doi:10.1007/s10295-015-1627-4

Miscevic, D., Mao, J. Y., Kefale, T., Abedi, D., Moo-Young, M., & Perry Chou, C. (2021). Strain engineering for high-level 5-aminolevulinic acid production in Escherichia coli. *Biotechnol Bioeng, 118*(1), 30-42. doi:10.1002/bit.27547

Srirangan, K., Liu, X., Westbrook, A., Akawi, L., Pyne, M. E., Moo-Young, M., & Chou, C. P. (2014). Biochemical, genetic, and metabolic engineering strategies to enhance coproduction of 1-propanol and ethanol in engineered Escherichia coli. *Applied Microbiology and Biotechnology, 98*(22), 9499-9515.


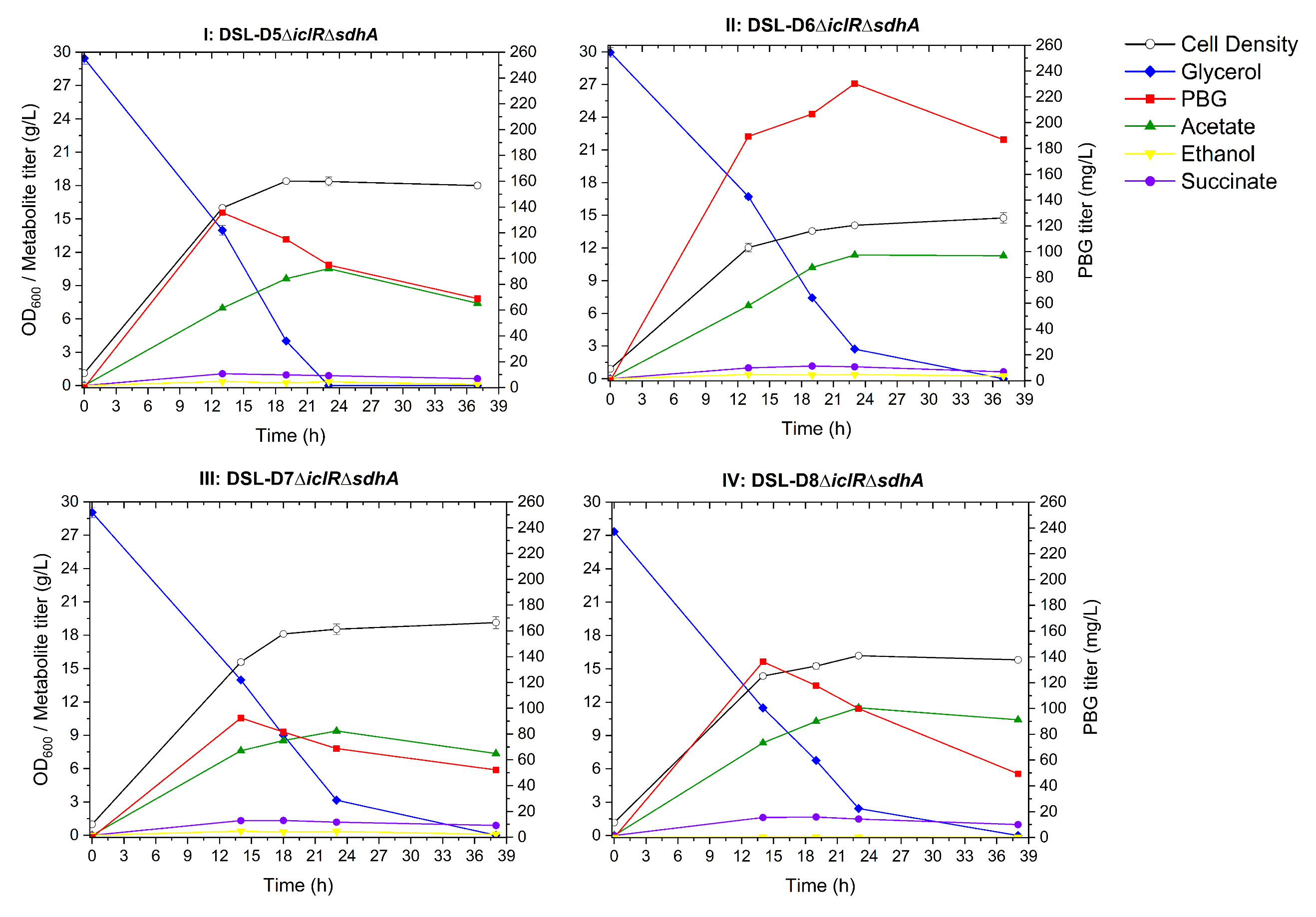

Supplement: Supplementary file 1 — Additional file 1: Table S1. DNA oligonucleotide sequences used in this study. Table S2. gRNA sequences targeting hemC for CRISPRi in this study. See Additiona file 1: Figure S1 for qRT-PCR results for select gRNAs. Table S3. Tabulated images of bioreactor cultivation samples under aerobic and microaerobic conditions. Table S4. Statistical analysis for comparing experimental data of PBG titers. Figure S1. Quantification of the relative hemC expression for select gRNAs using qRT‐PCR. All qRT‐PCR values are reported as means ± SD (n = 2). Figure S2. Bioreactor cultivation of DSL-D1∆iclR∆sdhA, DSL-D2∆iclR∆sdhA, DSL-D3∆iclR∆sdhA, and DSL-D4∆iclR∆sdhA for PBG biosynthesis under aerobic conditions. Time profiles of cell density (OD600), glycerol consumption and metabolite extracellular accumulation profiles are shown. (I) DSL-D1∆iclR∆sdhA, (II) DSL-D2∆iclR∆sdhA, (III) DSL-D3∆iclR∆sdhA, (IV) DSL-D4∆iclR∆sdhA. All values are reported as means ± SD (n = 2). Figure S3. Bioreactor cultivation of DSL-D5∆iclR∆sdhA, DSL-D6∆iclR∆sdhA, DSL-D7∆iclR∆sdhA, and DSL-D8∆iclR∆sdhA for PBG biosynthesis under aerobic conditions. Time profiles of cell density (OD600), glycerol consumption and metabolite extracellular accumulation profiles are shown. (I) DSL-D5∆iclR∆sdhA, (II) DSL-D6∆iclR∆sdhA, (III) DSL-D7∆iclR∆sdhA, (IV) DSL-D8∆iclR∆sdhA. All values are reported as means ± SD (n = 2). [file 40643_2021_482_MOESM1_ESM.docx]
